# Features of Fractal Conformity and Bioconsolidation in the Early Myogenesis Gene Expression and Their Relationship to the Genetic Diversity of Chicken Breeds

**DOI:** 10.3390/ani13030521

**Published:** 2023-02-01

**Authors:** Ivan I. Kochish, Evgeni A. Brazhnik, Nikolai I. Vorobyov, Ilya N. Nikonov, Maxim V. Korenyuga, Olga V. Myasnikova, Darren K. Griffin, Peter F. Surai, Michael N. Romanov

**Affiliations:** 1 K. I. Skryabin Moscow State Academy of Veterinary Medicine and Biotechnology, 109472 Moscow, Russia; 2 BIOTROF+ Ltd., Pushkin, 196602 St. Petersburg, Russia; 3 All-Russia Institute for Agricultural Microbiology, Pushkin, 196608 St. Petersburg, Russia; 4 School of Biosciences, University of Kent, Canterbury CT2 7NJ, UK; 5 Vitagene and Health Research Centre, Bristol BS4 2RS, UK; 6 Department of Microbiology and Biochemistry, Faculty of Veterinary Medicine, Trakia University, 6000 Stara Zagora, Bulgaria; 7 Department of Animal Nutrition, Faculty of Agricultural and Environmental Sciences, Szent Istvan University, H-2103 Gödöllő, Hungary

**Keywords:** chicken breeds, gene expression, myogenesis, genetic diversity, mathematical modelling, fractal analysis, bioconsolidation index

## Abstract

**Simple Summary:**

In the bodies of animals, including birds, gene expression leads to the synthesis of many proteins. To provide optimal cellular and organismal properties and functions, many genes should work in concert, reaching certain balanced relationships (or networks) between them and the intensities of their expression. Here, we studied the expression of several genes responsible for muscle formation and growth in chick embryos of diverse breeds belonging to various utility types. Using two mathematical (fractal) models and the respective indices, we showed that there are specific coordinated patterns of gene expression in the embryonic breast and thigh muscles. These patterns correlated with growth rate of chicks after hatching and depended on a utility type of the breeds studied. Overall, the proposed models contributed to an expanded understanding of the coordinated gene expression in early development and growth, providing additional characteristics of genetic diversity in chickens.

**Abstract:**

Elements of fractal analysis are widely used in scientific research, including several biological disciplines. In this study, we hypothesized that chicken breed biodiversity manifests not only at the phenotypic level, but also at the genetic-system level in terms of different profiles of fractal conformity and bioconsolidation in the early myogenesis gene expression. To demonstrate this effect, we developed two mathematical models that describe the fractal nature of the expression of seven key genes in the embryonic breast and thigh muscles in eight breeds of meat, dual purpose, egg and game types. In the first model, we produced breed-specific coefficients of gene expression conformity in each muscle type using the slopes of regression dependencies, as well as an integral myogenesis gene expression index (MGEI). Additionally, breed fractal dimensions and integral myogenesis gene expression fractal dimension index (MGEFDI) were determined. The second gene expression model was based on plotting fractal portraits and calculating indices of fractal bioconsolidation. The bioconsolidation index of myogenesis gene expression correlated with the chick growth rate and nitric oxide (NO) oxidation rate. The proposed fractal models were instrumental in interpreting the genetic diversity of chickens at the level of gene expression for early myogenesis, NO metabolism and the postnatal growth of chicks.

## 1. Introduction

Fractal analysis and fractional calculus are now widely used in all areas of science. including biology and biomedicine (e.g., [1,2,3,4,5]). The dynamics of biological systems can be understood mathematically using ordinary differential equations of integer and fractional orders as well as fractal dimension calculation [1,6,7]. In some cases, the fractional-order differential equations models appear to be more accurate representations of the observed biological events compared to the integer-order models [7]. The applications of fractal analysis, fractional-order differential equations and fractal dimension-based methods are thus becoming increasingly important in biology. A biodiversity assessment using various methods, including mathematical models, is an important area in life, earth and agricultural sciences [8,9,10,11,12]. The evaluation of genetic diversity between breeds is crucial for creating innovative breeding and production methods in poultry as well as for preserving genetic resources [13,14,15,16,17].

A fractal object is characterized by the property of scale invariance, or scaling, i.e., the ability to reproduce the object when the scale changes. In addition, the fractal dimension can be either integer or fractional. Natural fractals are considered on a certain scale as preserving the property of self-similarity [18,19,20]. At the population level, it was shown that the power-law dependence of species diversity on the size of the habitat area implies the self-similarity of the community structure. Particularly, the theory of island biogeography was built on the basis of power laws [21]. It can be assumed that if the community structure is self-similar [21] and its members exhibit morphological characteristics of self-similarity, then fractal properties should also manifest themselves at the level of regulation of biological processes. From this, it can be assumed that for each stable system (species or breed), there should be rules of internal organization that are of a fractal nature and characterize its individual features.

As a result of gene expression in living organisms, including birds, numerous proteins are synthesized that always have a well-defined chemical formula prescribed in the corresponding genetic codes. When a protein or protein complex is formed due to the expression of several genes, certain balanced relationships should also be established between the expression intensities of these genes. Otherwise, the monomers from which the protein is assembled will either not be enough, or there will be an excess of them. The optimal, energetically favorable situation corresponds to such a ratio of gene expression products, in which the target protein or protein complex is produced in each step of biosynthesis without loss and in full. Based on general observations of the morphogenesis of living organisms, it was found that most of the quantitative ratios among the organs of animals and plants correspond to geometric fractal sequences (or power series) [20] or, when taking logarithms of phenotypic data, to arithmetic fractal series [22,23].

Previously, it was found that the oxidation rate of nitric oxide (NO) to nitrate in a chick embryo correlates with the postembryonic growth rate (GR) and, therefore, this parameter can, to a certain extent, be associated with the productivity of meat chickens [24,25,26,27]. Other studies have also shown the importance of NO in embryogenesis and, in particular, myogenesis (e.g., [28,29]) by regulating the growth of myocytes, satellite cells and muscle fibers [30,31,32,33,34,35]. Cazzato et al. [28] explored the expression of several important genes that regulate the formation of skeletal muscle during the earliest embryogenesis stages, including myosin (*MYH1*; e.g., [36]) and other associated genes. NO synthase inhibitor and NO donor (NOD) compounds had an impact on the myogenesis gene expression [28]. In a preliminary study [25], the myogenesis gene expression in the muscle tissue of 14-day-old (E14) embryos was compared with a greater NO oxidation rate in the Mini Meat and White Cornish (WC) breeds on the one hand and a lower rate in the Plymouth Rock White (PRW) and layers (LR) on the other. It was previously suggested [25] that in the embryos with a higher degree of NO oxidation, the expression of the myogenesis factor 5 (*MYF5*) gene in the breast and thigh muscles was, respectively, 2^4^–2^7^ and 2^4^–2^10^ times lower than those in embryos with a lower oxidation rate. In addition, the expression of the myogenic differentiation 1 (*MYOD1*) gene in the thigh muscles was 2^3^ to 2^6^ times lower in embryos with a higher rate of oxidation. There were no significant differences in the expression of the *MYH1*, myogenin (*MYOG*) and myocyte proliferation factor 2c (*MEF2C*) genes. In this regard, further and more detailed studies of the expression patterns of early myogenesis genes in various chicken breeds are required to characterize their genetic diversity. The latter forms the basis of poultry genetic resources [37,38,39,40,41,42] and embraces breeds of several main types of artificial selection targeting and utility, including meat, dual purpose, egg and game breeds [13,14].

In former studies, we attempted to apply mathematical modeling, including derivation of fractal structures and indices, to assess microbiome diversity and growth dynamics in chickens [22,23,43,44]. Changed fractal bioconsolidation patterns were discovered among communities of microorganisms in the chicken intestines due to the use of various feed additives. Therefore, it seems relevant and important to search for fractal patterns and use them to assess such a genetic system as the myogenesis gene expression at the stage of chicken embryogenesis. Inspired by recent practical implementations in modeling complex processes from various domains, first of all, fractional-order models in biology and biological applications, and based on our own preliminary work, we aimed here to develop methods of deriving fractal models and indices for assessing the early myogenesis gene expression in various chicken breeds. Using these models, it would be desirable to show that breed diversity manifests itself not only at the external phenotypic level, but also at the genetic system level in the form of different values of the fractal coefficients of myogenesis gene expression. It would also be important to establish the presence of a possible relationship between the fractal indices of gene expression in the embryonic muscles, GR and the levels of NO metabolites.

## 2. Materials and Methods

### 2.1. Experimental Birds and Raw Data Generation

Fertilized eggs of eight chicken breeds (Table 1) obtained from Genofond LLC (Sergiev Posad, Moscow Oblast, Russia) were used in the experiments as follows: Broiler (BR; Smena 8 cross), WC (Smena 8 paternal stock), PRW (Smena 8 maternal stock), Yurlov Crower (YC), Brahma Buff (BB), Orloff Mille Fleur, LR (Hisex White cross) and Uzbek Game (or Kulangi). These breeds represented four main types of utility, i.e., meat, dual purpose, egg and game [13,14]. All experimental groups contained 70 eggs per breed subject to incubation and subsequent chick hatch.

To produce molecular data, three biological replicates (samples) per breed were employed, and there were three technical replicates per sample and for each type of muscle tissue. Within one breed/tissue, the difference between replicates was not statistically significant (*p* > 0.05). The standard error between replicates did not exceed 5%. Muscle tissue homogenates (breast and thigh) from E14 embryos were produced using a tissue grinder for further assessment of the relative differential gene expression (DGE). The latter was examined using quantitative real-time PCR and sets of primer pairs published elsewhere (e.g., [28]) for the following seven myogenesis genes: myostatin (*MSTN*), growth hormone receptor (*GHR*), *MEF2C*, *MYOD1*, *MYOG*, *MYH1* and *MYF5*. The housekeeping gene of the TATA-binding protein (*TBP*) was employed as an internal DGE control. Expression of the tested genes was calculated as fold change (FC) values relative to the control gene expression and according to the Livak–Schmittgen method [45,46]. The NO oxidation state was assessed as described elsewhere [47,48] (see also more details in Supplementary Information S1). Briefly, 7-day-old (E7) embryonic homogenates were prepared by processing the contents of a shell-less egg in a glass homogenizer. The content of NO metabolites in the embryo samples was determined using an enzyme sensor. The rate of NO oxidation to nitrate was estimated by the ratio of nitrate and NOD compounds concentrations as follows: nitrate/(NOD + nitrate) × 100%. All proper measures were undertaken to eliminate any batch effect and reduce the influence of any other experimental factors, both technical and biological, in the course of egg incubation, sample collection and subsequent analyses [49].

### 2.2. Statistical and Mathematical Analyses

For statistical processing of the obtained data, the mean values and their standard errors (M ± m) were calculated. The significance of differences between the compared indicators was assessed using Student’s *t*-test; differences were considered statistically significant at *p* < 0.05. Subsequent statistical processing of the experimental data was carried out using the RStudio program (Version 2022.07.1+554) [50]. The raw FC data were normalized using log_2_ calculation. For regression analysis (e.g., when deriving fractal indices), ascending gene expression rank numbers (*N*) were taken as the independent variable, and the transformed |FC − 1| values served as the dependent variable. A linear regression model for gene expression data (Table 2) across the breeds by muscle type was computed in R using the lm() function [51]. Linear regression model plots were built using the ggplot2 package for R [52,53,54]. To assess the normality of the distribution of quantitative traits, the Shapiro–Wilk test was employed using the shapiro.test() base function for R. Since the data did not have a normal distribution, correlation analysis was performed using the Spearman test and the cor() base function for R. Data visualization was generated using the corrplot package (version 0.90) for R [55]. GR was estimated from the rate of body weight gain over a 4-week period. Two GRs were obtained, at two (GR2wk) and four (GR4wk) weeks, by dividing the respective body weight values at 2 or 4 weeks by body weight at day-old.

The fractal pattern of gene expression was assessed in two ways. In the first model, this was done using a power-law relationship between the FC value and the number *N* of gene ranks involved in the overall myogenesis process, which can be written as:

(1) $$\left|\mathrm{FC}-1\right|\left(N\right)=a+K\times e^{N}$$

where *N* is a predictor variable conforming to the rank number for the genes under consideration; |FC − 1| is a dependent variable on *N*; *a* is an intercept (i.e., a variable that specifies the same offset for all |FC − 1| values and corresponding to the initial curve growth point) and *K* is a coefficient reflecting the slope of the graphical function and determining the growth characteristic for |FC − 1|. Conformity of the gene expression function to Equation (1) is a diagnostic sign of a fractal object [18,19,20]. At the same time, the fractal dimension can not only be an integer, but also fractional, which in our case justifies the use of the Euler’s number in Formula (1). In the second model, we used the conventional formula for calculating the fractal dimension:

(2) lg(|FC−1|)=D×lg(N)+lg(c)

where *D* is the coefficient of fractal dimension, and *c* is a variable that specifies the offset of the starting point along the *y*-axis.

## 3. Results

### 3.1. Relative Differential Gene Expression Data

According to the relative DGE data in the muscle tissues of E14 embryos (Table 2), myogenesis genes were found to be up- and downregulated in the eight different breeds. For instance, YC exhibited noticeably higher FC values for the *MEF2C* gene expression in both the breast and thigh muscles while UG was characterized by lower FC values. Using this data, however, it was not possible to directly detect any distinct trend or pattern in the DGE levels across the examined breeds (Table 2). Each breed appeared to have its own unique combination of particular up- and downregulated genes. In this respect, two mathematical (fractal) models have been proposed to analyze the FC datasets for the breast and thigh muscles and are outlined below.

### 3.2. Fractal Analysis of Myogenesis Gene Expression Structure in Various Chicken Breeds

To establish the first mathematical model, we proceeded from the fact that a complex of genes with a different magnitude of activity is responsible for a certain ongoing biochemical or physiological process. Since fractals are characterized by a power-law dependence [18], we therefore assumed that this should also be traced in the regulation of gene network. Consequently, elements of the fractal analysis approach were employed to develop the first model. Within the framework of this model, we approximated the dependence of the transformed FC values on the rank number *N* of myogenesis genes using a power function, determined the slopes of this function (*K*) in various breeds and found myogenesis gene expression indices (MGEIs) as ratios of the *K* coefficients for the breast and thigh muscles. In addition, breed fractal dimensions were identified for gene expression in two types of muscle tissue, and on that basis, an integral myogenesis gene expression fractal dimension index (MGEFDI) was derived.

#### 3.2.1. FC Value Transformation and Gene Ranking

Let us now consider the FC levels in the seven genes studied in the breast and thigh muscles of chick embryos (Table 2). As we can see from the primary DGE data in this experiment, genes can be either upregulated or downregulated. DGE levels of these genes are symmetrical with respect to the housekeeping gene *TBP*, whose expression level was taken as 1. In this regard, the goal of further FC transformation was to get away from the negative values of downregulation and transfer all values to the positive semiaxis of the numerical straight line. Such an assumption seems, in our opinion, justified. For instance, if the expression of the housekeeping gene is set to 1, the DGE of an upregulated gene is 6 and that of a downregulated gene is −4, then the magnitude of the DGE change for these genes is the same and equals 5. It is generally accepted that an upregulated gene stimulates the synthesis of one protein and a downregulated gene will conform to a reduced production of another protein, and this will be true if we talk about certain known simple biochemical processes. If we consider the functioning of these genes in concert and on the scale of the whole organism networks, it may turn out that the effects of up- or downregulation of these genes will be quite ambiguous. Along with the gene downregulation, there may also be an activation of some other gene/process. For example, the downregulation of the *MYH1* gene was mostly inversely associated with the growth of the thigh muscles in E14 embryos of the BR, WC, PRW, OMF and UG breeds in our experiment (Table 2).

Hence, in order to assess the effects of the considered genes comprehensively, the appropriate transformations were made. To do this, we removed the consideration of the *TBP* gene and negative values of key myogenesis genes, for which we reduced the DGE data to zero symmetry by calculating the difference between FC and one and, then, converting it to modulus, i.e., |FC − 1|. It seems that such mathematical treatment of both up- and downregulated genes’ data most likely allows the simplification of this genetic system and the consideration of genes with different directions of activity simultaneously based only on their magnitude of expression. This approach has also been used in some other studies. For instance, data transformations without attention to up- and downregulation levels can be found in refs [56,57] where the results were plotted as relative FC values without regard to their sign and with a minimum value of zero. Indeed, the direction of gene effects (up or down) was neglected in our study after the transformation, but the magnitude of these effects was preserved. The produced numerical values were ranked in ascending order of the new (after transformation) FC values, i.e., |FC − 1| values (Tables S1 and S2).

#### 3.2.2. Approximation of the Dependence of FC Level on the Rank of Genes

**Figure 1:**
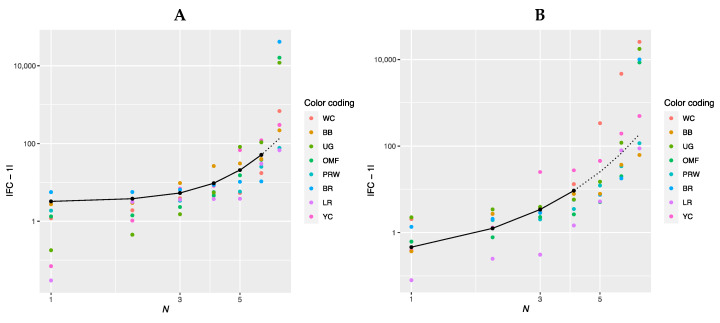
Log–log plots of a linear regression model for gene expression data (Table 2) in the eight breeds by muscle type (shown by a solid line). (**A**) Breast muscles (*N* = 1…6); |FC − 1|(*N*) = 0.12962 × exp(*N*); adjusted *R*^2^ = 0.5485. (**B**) Thigh muscles (*N* = 1…4); |FC − 1|(*N*) = 0.16990 × exp(*N*); adjusted *R*^2^ = 0.4220.

When examining the data for gene ranks in Tables S1 and S2, no regularity can be found in the gene expression level. Each breed has its own combination of genes, which will be discussed below when plotting regression curves. To identify rank dependencies, we produced a graph in log–log plots (Figure 1) where the gene rank was considered an independent variable and was presented along the abscissa while the FC level was a dependent variable along the ordinate. On the chart in Figure 1A (for the breast muscles), the solid line conforms to the genes of ranks 1 to 6 (*N* = 1…6). When plotting the graph in Figure 1B (for the thigh muscles), the genes of ranks 1 to 4 (*N* = 1…4) were taken into consideration. At the same time, the genes of rank 7 (Figure 1A) and ranks 5 to 7 (Figure 1B) were not explained by the corresponding curves and did not conform to the obtained equations.

In Figure 1A,B, the regression line is shown as a solid line. It is defined for the breast muscles by the regression equation |FC − 1|(*N*) = 0.12962 × exp(*N*), where *N* is the ordinal number of the gene rank from 1 to 6 and FC is the FC level of a gene, whereas its interpolation is indicated by a dotted line. For the thigh muscles, the regression model is described accordingly by the equation |FC − 1|(*N*) = 0.16990 × exp(*N*). The Pearson’s correlation coefficient between predicted and actual values was 0.64 for the breast muscles (at *p* < 0.001) while that for the thigh muscles was 0.46 (*p* < 0.01). Figure 1A,B show that the FC values have an exponential growth. Rank 7 breast muscle genes have significantly higher FC values and do not fall under the explanation of the growth exponent of the above equation; for the thigh muscles, genes of the 5th, 6th and 7th ranks do not fall under this explanation. The regression equation for the breast and thigh muscles, respectively, can also be rewritten as follows: |FC − 1|(*N*) = 0.12962 × *e^N^* and |FC − 1|(*N*) = 0.1699 × *e^N^*, where e ≈ 2.718 (Euler’s number).

Thus, the above equations suggest that gene expression can be attributed to genetic systems and other biological phenomena that develop exponentially and in which there is a feedback (e.g., an increase in the mass of animals, a growth in the number of a species/population size, etc.). In addition, one can argue about the fractal pattern of gene expression that is expressed in a power-law relationship between the FC value and the number of genes involved in the overall process (Formula (1)), which is a diagnostic feature of a fractal object [18,19,20].

Transforming the graphs in Figure 1 and deriving a new one shown in Figure S1, in which genes belonging to the same breed are connected by lines, broiler breeds (i.e., WC, PRW and BR) had a less flat line while LR, YC and OMF had the least sloping line. Thus, if we draw an approximation line using |FC − 1|(*N*) points for each breed, its slope angle will characterize the pattern of breed myogenesis gene expression. The slope angle is the rate of increase in the FC level depending on the number of genes under consideration, i.e., equaling to the ratio |FC − 1|/*N*.

As noted above, the description of the exponential increase in expression in the breast muscles does not include the gene of the 7th rank. Therefore, to approximate the expression within each breed, we will further take only six genes from the first six ranks. To approximate the |FC − 1| values for the thigh muscles within breeds, we will further use only genes from the first four ranks, since their value can be described by the above regression equation (i.e., |FC − 1| = 0.16990 × exp(*N*)). With this reduction in the number of ranks (genes) under consideration, the plots can be presented in the conventional rather than log–log coordinates (Figure S2).

#### 3.2.3. Determining the Slope of the Function |FC − 1| = f(N)

**Figure 2:**
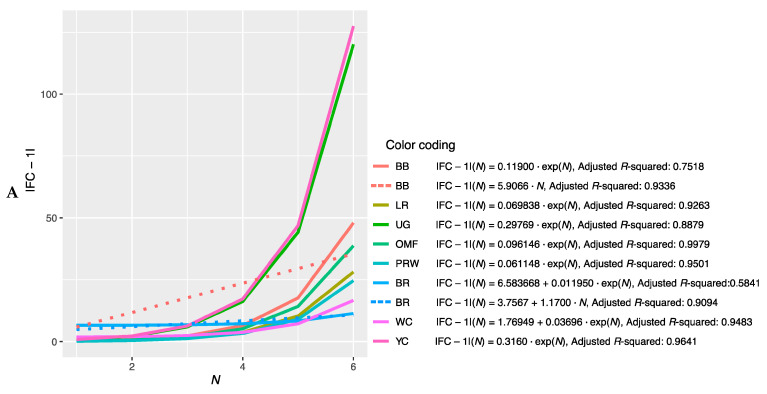

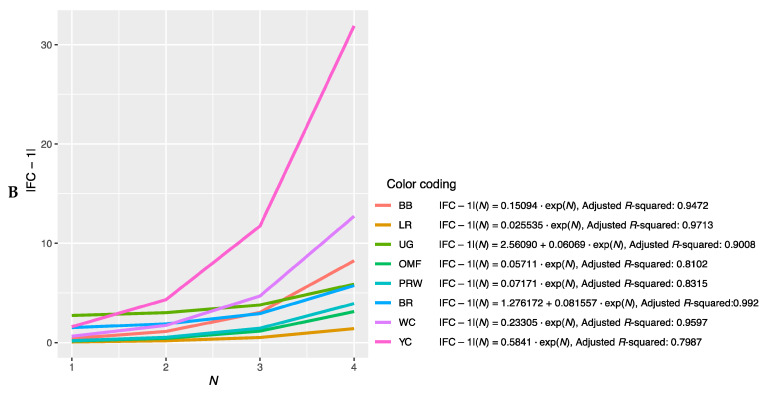
Combined plots for breed-specific regressions |FC − 1| = *f*(*N*) in the different breeds by muscle type. (**A**) Breast muscles; (**B**) thigh muscles.

Further, we combined the graphs for single-breed regressions |FC − 1| = *f*(*N*) (Figure S2A–P) and derived new plots shown in Figure 2. The dependences of |FC − 1| in the breast muscles of different breeds on the number of genes considered in order of rank are presented in Figure 2A and described by the respective regression equations. The dotted line represents additional linear regression models for the BB and BR breeds. On this combined plot, one can distinguish a group of three broiler breeds of meat (meat-egg) type, i.e., WC, BR and PRW, that have graphically similar broken curves for the dependence |FC − 1| = *f*(*N*) located near each other and at the lowest level relative to other breeds. At the same time, PRW, being the dual purpose breed, still deviates from the two true breeds of meat type, WC and BR, towards the egg-type (LR) and other dual purpose (OMF and BB) breeds. The graphic dependences |FC − 1| = *f*(*N*) for the YC dual purpose and UG game breeds go up the most. On the graph for the thigh muscles (Figure 2B), one can observe different patterns of |FC − 1| = *f*(*N*). At the lowest level is LR; WC and especially YC go above other breeds while the other breeds take intermediate positions.

Note that for the intrabreed approximation (Figure 2 and Figure S2), we used Formula (1), where *a* is a variable ranging from 0 (in PRW, YC, BB, OMF, LR and UG) to 6.584 (in BR). This variable is responsible for the level of the initial shift of the curve, i.e., the level of its initial rise, below which the subsequent levels |FC − 1| of genes will not go down and can only grow. In this case, GR of the function is determined by the coefficient *K*, the minimum value of which equals 0.012 (BR) and the maximum value is 0.316 (YC). Thus, it can be argued that the signals of gene expression for breed types also differ in the rate of increase in the function determined by Equation (1). In other words, by the value of the slope angle (*K*), one can judge the tightness of the relationship of the genes involved in the overall coordinated process of early myogenesis in chick embryos.

#### 3.2.4. Deducing the Myogenesis Gene Expression Index

Characteristics of the studied breeds in terms of phenotypic traits (body weight in dynamics), metabolic features (indicators of NO metabolism) and *K* coefficients, reflecting the levels of myogenesis gene expression in the breast and thigh muscles, are presented in Table 3. Hereby, indicators of metabolic levels and breed types were also considered as nominative data (i.e., “high” vs. “low” and “meat” vs. “no”, respectively), and PRW, a maternal line of the broiler cross, was assigned to the meat type in this comparison. As you can see, the *K*(br) coefficient values from 0.012 to 0.061 were typical for the breast muscles of broiler breeds and those from 0.070 to 0.316 for other breeds. For the thigh muscles, the *K*(th) coefficient values for broiler breeds varied from 0.072 to 0.233 and those for other breeds from 0.026 to 0.584. These comparisons testified that the broiler breeds differed from other breeds in the myogenesis gene expression pattern in the breast muscles towards lower *K*(br) coefficient values. In the thigh muscles, the distribution of expression had a more complex pattern. As for the indicators that determine the levels of NO metabolites, it can be seen that there were apparently no special patterns (correlations) for the breed belonging to one or another type of utility (category) and for the variation of *K* coefficients’ values. An exception in this regard, however, was the meat-type breeds BR and WC, as well as the UG game breed, which were characterized by a high degree of NO oxidation (~97–98%).

From this dataset, we can examine individual indicators, e.g., the *K* coefficient values in the breast muscles in comparison with the body weight dynamics in the eight chicken breeds studied (Table 3 and Table S3). Note that the obtained *K* values and the breed characteristics of body weight, apparently, come from a general population that does not have a normal distribution. Similarly, other physiological (metabolic) parameters did not seem to have a normal distribution (Table 3). In this regard, to check the normality of data distribution, the Shapiro–Wilk test was used, which reliably showed an abnormal distribution of values for each indicator (at *p* < 0.05). Based on this, Spearman’s rank correlation test was further applied to search for significant relationships (as compared to Pearson’s correlation; Table S4). Testing was carried out on primary gene expression data as is and after their normalization by taking the natural logarithm. Normalization slightly improved the data, brought it closer to a normal distribution and also increased the correlation coefficient values and their significance (Table S4). Herewith, in addition to the *K* coefficients in the breast (*K*(br)) and thigh (*K*(th)) muscles, an integral indicator was introduced, which we designated as the myogenesis gene expression index (MGEI):

MGEI = *K*(br)/*K*(th) (3)

To establish the relationship between the obtained coefficients and different types of categories (NOD, nitrate, NO oxidation and breed type), the ANOVA test was carried out. There was an association between the breed type (meat vs. others) category and the *K*(br) coefficient (*p* = 0.09 before coefficient normalization and *p* = 0.0244 after normalization). In addition, a significant relationship was found between the breed type category and GR2wk and GR4wk (*p* < 0.001). In other cases, significant associations could not be established (Table S4).

A comparison of *K*(br) and *K*(th) coefficients and their ratio *K*(br)/*K*(th) depending on the type of breed utility is shown in Table S5. It can be seen that the coefficients are mutually inversely proportional for different breed types, and thus the introduction of a new integral indicator MGEI in the form of the ratio *K*(br)/*K*(th) seems justified. This ratio, in our opinion, adequately and well characterizes the processes of early embryogenesis in chickens and can serve as an integral indicator of myogenesis gene expression in both muscle types. In addition, when using this index, the correlation coefficient also improved slightly (Table S4). It is also worth noting that when ranking by different coefficients, i.e., *K*(br), *K*(th) and MGEI, a cluster-like pattern was observed for certain breed types in Table S5. Only the BB and YC breeds did not have a clear position.

The next step was to transform the data from Table S5 by sorting the breeds by MGEI values and add the FC scores for the *GHR* gene, an important regulator of growth (Table S6). In principle, it can be stated that the ranking by increasing MGEI values conformed to the grouping of breeds according to their utility type: meat > dual purpose > egg > game. At the same time, there is no clear relationship between breed type and single gene FC values (i.e., *GHR* in the breast muscles in this example; Table S6).

Further, one can try to link the myogenesis gene expression in E14 embryos with the GR of chicks (due to the growth of the skeleton and muscles, primarily the breast and thighs) during the first 2 (GR2wk) and 4 (GR4wk) weeks of life (Table 3, Table S7 and Table S8). In terms of GR, we had a clear relationship between the increase in body weight and the utility type that progressed from the egg breed to meat breeds. However, there was again no clear relationship between breed GR and single gene FC (*GHR* in the breast muscles in this example; Table 3 and Table S8).

**Figure 3:**
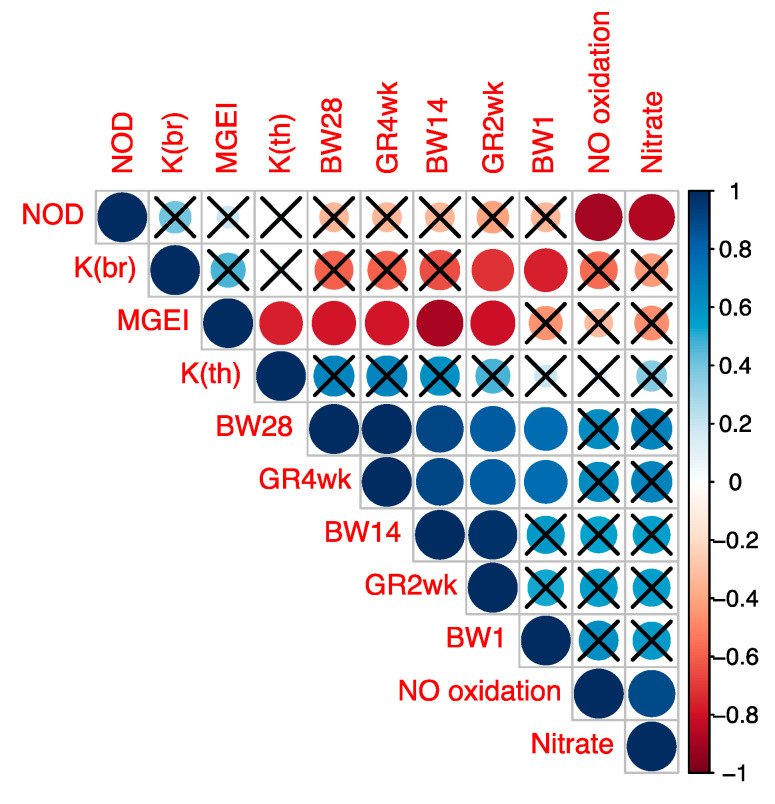
Spearman’s correlation coefficients between the studied indicators. Abbreviations: BW1, BW14 и BW28, body weight at 1, 14 and 28 days; GR2wk and GR4wk, growth rate for 2 and 4 weeks; NOD, content of NO donors; Nitrate, content of nitrate; NO oxidation, rate of NO oxidation to nitrate; *K*(br), *K* coefficient in the breast muscles; *K*(th), *K* coefficient in the thigh muscles; MGEI, myogenesis gene expression index expressed as the ratio *K*(br)/*K*(th). Significant correlations are not crossed out with the sign «×» (*p* < 0.05).

Subsequently, we determined the correlation between MGEI on the one hand and growth factors (GR2wk, GR4wk) and other studied indicators on the other. The visualization of correlations between these and other indicators is given in Figure 3 and was based on the Spearman’s correlation coefficient values (Table S9) and their corresponding *p*-values (Table S10). From the data in Figure 3, significant correlations were identified between MGEI on the one hand and growth traits (BW14, BW28, GR2wk and GR4wk) on the other. In addition, there was a significant correlation between *K*(br) and the two growth indicators (BW1 and GR2wk). Significant correlations were also found within the group of growth traits (BW1, BW14, BW28, GR2wk and GR4wk) and the group of NO metabolism indicators (NOD and nitrate content and NO oxidation rate). These correlations confirm the significance of the observed relationships between the parameters and indices of myogenesis, growth and NO metabolism examined in the present study. In general, according to the results of the correlation analysis, we validated the specific illustrative model developed by us and described above by regression curves that demonstrates the fractal pattern of gene activity in birds of different breeds and linking such physiological (phenotypic) indicators as GR and productivity with genetic features.

#### 3.2.5. Defining Fractal Dimension D

The analysis of fractals can ultimately be linked to the derivation of fractal dimension *D*. If the data has fractal properties, we can approximate it with an exponent. Herewith, a curve will be obtained, and when taking a logarithm (in ordinary coordinates), it will be a straight line described by Equation (2) while its slope angle will conform to the fractal dimension *D*. The fractal dimension *D* is determined as the tangent of the slope of a plotted curve [58]. Based on this, we evaluated the possibility of calculating the fractal dimension *D* by simplifying Formula (2) and removing the variable *c* from it to obtain the following equation found also in other literature sources:

*D* = lg(|FC − 1|) / lg (*N*) (4)

Accordingly, the fractal dimension *D* was defined here as the slope of the lg (|FC − 1|) depending on lg (*N*). To implement this, regression plots of lg (|FC − 1|) vs. lg (*N*) were built based on the principle of choice of gene ranking and their number, i.e., 6 and 4 for the breast and thigh muscles, respectively (Figure S3).

For a few breeds, however, it was necessary to correct the mathematical models for the dependencies of lg (|FC − 1|) on lg (*N*). Accordingly, based on the nature of each individual curve, we additionally manually selected the composition of gene ranks in a particular breed in order to describe more adequately the breed-specific fractal dimension patterns for the observed myogenesis gene expression and deduce more optimal fractal dimension coefficients *D* (e.g., Figure S3B2b,C1b,E1b,F1b). The resulting fractal dimension coefficients in the breast (*D*(br)) and thigh (*D*(th)) muscles are presented for the eight breeds studied in Table 4. The fractal dimension coefficient *D*(br) ranged from 0.400 (BR) to 4.104 (YC) and *D*(th) from 0.632 (UG) to 11.211 (WC).

Just as in the case of the *K*(br) and *K*(th) coefficients, it can be seen, to a certain extent, that the *D*(br) and *D*(th) coefficients tend to be inversely proportional for different breed types. Accordingly, similar to the derivation of MGEI from the *K* coefficients for the breast and thigh muscles (Formula (3)), we proposed to define an additional index based on the *D* coefficients and call it the myogenesis gene expression fractal dimension index (MGEFDI):

MGEFDI = *D*(br)/*D*(th) (5)

MGEFDI values (Table 4) ranged from 0.115 to 0.475 in the broiler breeds (BR, WC, PRW), 1.215 to 2.634 in the dual purpose breeds and LR, and it was as high as 5.915 in UG. When comparing *D*(br), *D*(th) and MGEFDI values with *K*(br), *K*(th) and MGEI, one can notice certain common trends and patterns of these indicators depending on the types (categories) of breeds (Table S11).

When evaluating the linear relationship between the fractal dimension coefficients *D* and BW28 (see Supplementary Information S2), it can be noted that *D*(br) is linearly related to the body weight of birds (at *R*^2^ = 0.456; Figure S2-1A in Supplementary Information S2). The manual correction of *N* values to determine the straightest curve sections for the BB, OMF and PRW breeds significantly increased the predictive power of this model (*R*^2^ = 0.816; Figure S2-1B in Supplementary Information S2). On the other hand, the fractal dimension coefficient *D*(th) does not appear to be linearly related to BW28 (Figure S2-1C,D in Supplementary Information S2).

### 3.3. Fractal Portraits and Fractal Bioconsolidation Index of Gene Expression

When considering the second fractal model, we proceeded from the assumption that, under the optimal mode of gene expression in birds, the ratio of expression intensities should correspond to log arithmetic series and the gene expression bioconsolidation index equal to one. Accordingly, we developed a method for calculating this fractal bioconsolidation index that could be used to explore if the diversity of breeds manifests itself not only at the external phenotypic level, but also at the genetic system level in the form of different values of the bioconsolidation index of myogenesis gene expression. Additionally, the presence of a possible relationship between the indices of gene expression bioconsolidation in the embryo muscles and the levels of NO metabolites was examined.

If considering an example of the myogenesis gene expression for the breast muscles of WC embryos and using the expression intensities presented by FC values for at least three genes (e.g., #1, #4 and #2; Table 2 and Table S12), there is a log arithmetic series of three numbers {1; 1 + 1.91 = 2.91; 2.91 + 1.98 = 4.89}. Consequently, we assume that these genes are included in the respective expression fractal. For the subsequent detection of the arithmetic sequence of gene expressions formed by the numerical indicators of FC, it is necessary to convert them according to the following formula:

(6) $$\mathrm{LG}=\{\begin{array}{c} -\log_{2}\left(\mathrm{FC}\right),\mathrm{if}\;\mathrm{FC}\geq 0\\ \log_{2}\left(\mathrm{FC}\right),\mathrm{if}\;\mathrm{FC}<0 \end{array}$$

where LG is the transformed indicator of gene expression, and FC is the numerical indicator (fold change) of gene expression (from Table 2). For example, for the breast muscles in WC, we have: LG*_MSTN_* = −log_2_ (4.89) = −2.29, LG*_MYF5_* = log_2_ (−685) = 9.42 and so on (Table S13).

**Figure 4:**
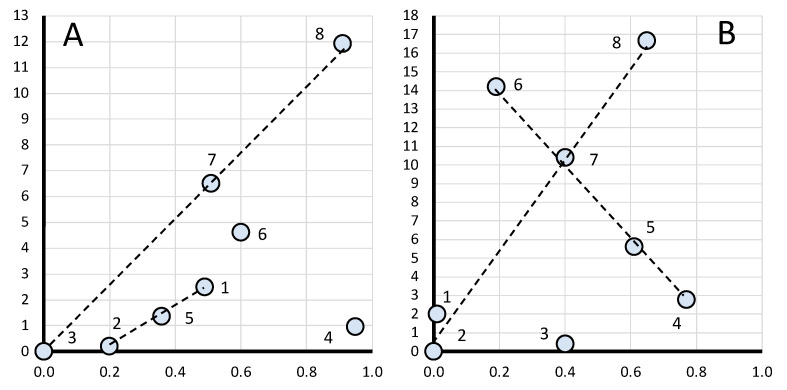
Fractal portraits of gene expression in the breast (**A**) and thigh (**B**) muscles of WC embryos. The *X*-axis shows the fractional parts of the shifted gene expression levels, and the *Y*-axis shows the full values of the shifted gene expression levels (Table S13). The numbers near the dots correspond to the gene numbering in Table S13. The dotted lines show that the corresponding genes belong to gene expression fractals, reflecting the fractal bioconsolidation of these genes.

For the visual detection of expression fractals, it is necessary to plot a fractal portrait of gene expressions. The fractal portrait of expressions is a field of dots depicting the expression of each gene encoded by the location coordinates of the dot in the portrait. Before constructing a fractal portrait of gene expression, it is necessary to transform the initial FC data of gene expression (Table 2) using Formula (6) and the number conversion algorithm presented in Table S13 and described below. Constructing a fractal portrait of gene expression include (i) the LG values from Table S13, (ii) the Y- and X-coordinates of the point that displays the expression of this gene in the portrait and (iii) the point placed on the portrait in accordance with these coordinates. For example, to transform LG values (Table S13), one should first search for a gene with the lowest expression level: for the breast muscles, this is the *GHR* gene (−2.49). After that, the value of the lowest gene expression is subtracted from the expressions of the remaining genes. As a result, we get altered gene expressions that are used as Y-coordinates (Table S13). X-coordinates are deduced by taking the fractional part of the decimal number that determines the Y-coordinate of gene expression. For instance, the shifted expression intensity of gene #5 (*MYOG*) is 4.6. Therefore, the point corresponding to this gene should be placed on the portrait with coordinates: Y = 4.6; X = 0.6 (Table S13). Using this computational algorithm, two fractal portraits were built for the genes of the breast and thigh muscles of the WC breed (Figure 4A,B), on which the points are located in accordance with the calculated Y- and X-coordinates.

The above formulated definition of a gene expression fractal is based on the concept of log arithmetic series of gene expression values. For example, the expressions of genes #2, #5 and #1 in the WC breast muscles form an arithmetic series and are also located on the same straight line in the fractal portrait (Figure 4A). Based on this, we can formulate in a more general form the condition that genes belong to a gene expression fractal. A characteristic of fractal bioconsolidation of gene expression and belonging of genes to an expression fractal is the location of the corresponding (at least three) genes on straight dotted lines.

The fractal portraits of gene expression in the breast muscles of WC embryos (Figure 4A) show that expression fractals are formed by genes #1, #2, #3, #5, #7 and #8 (six genes), i.e., this group of genes demonstrates fractal bioconsolidation. In the fractal portrait for the thigh muscles (Figure 4B), expression fractals are composed of genes #2, #4, #5, #6, #7 and #8 (six genes), which also conform to fractal bioconsolidation. Herewith, the location of points on the fractal portrait on one straight line corresponds to the condition for the formation of an arithmetic series of these numbers. For example, for the FC values of genes #2, #5 and #1 in the breast muscles of WC embryos, an arithmetic series of three LG values is obtained (Formula (6); Table S13): {0.20; 0.20 + 1.16 = 1.36; 1.36 + 1.13 = 2.49}. Therefore, both the arithmetic series and the arrangement of points on the same straight line in the fractal portrait are confirmatory signs of bioconsolidation of gene expression in bird organisms. The fractal portraits of gene expression for the other breeds studied are shown in Figure S4.

The optimal development of embryos and chicks and their accelerated body weight gain can be associated with the closeness of the proportions of gene expressions to log-arithmetic series. In other words, we assume that the more genes are included in gene expression fractals, the more favorable is the internal organization of coordinated gene expressions in the body of embryos and chicks and the faster and more fully they develop. Given that genetic processes in the breast and thigh muscles occur simultaneously and in parallel, it was proposed to calculate the bioconsolidation index of gene expression (*Ind*) using the following formula:

(7) $$Ind=\frac{\sqrt{N_{B}\cdot N_{T}}}{N_{G}}$$

where *N_B_* and *N_T_* are the number of genes included in the gene expression fractal in the breast and thigh muscles of the embryos, respectively, and *N_G_* = 8 is the total number of controlled genes in the body of the embryos. For the WC breed, we obtain according to Figure 4A: $Ind=\frac{\sqrt{6\cdot 6}}{8}=0.75$.

The *Ind* value can vary from zero to 1. The minimum *Ind* value (0) will theoretically correspond to the absence of genes that form expression fractals in the breast and/or thigh muscles of the embryos. The maximum *Ind* value (1) is possible when all eight genes in both the breast and thigh muscles form the corresponding fractals.

The results of calculating the *Ind* index for the chicken breeds studied, along with their other phenotypic indicators, are shown in Table S14. Based on the data of Table S14, it can be seen that there was a positive but lower correlation of the bioconsolidation index with egg weight (*r* = 0.48) and a high correlation with chick weight (*r* = 0.73…0.77) and total NO metabolites (*r* = 0.89). A correlation was also found between the sum of NO metabolites and chick weight (*r* = 0.56…0.72) and egg weight (*r* = 0.45).

## 4. Discussion

As a result of its capacity to ensure superior modeling and the control of dynamical systems, fractional calculus is now employed in many interdisciplinary applications, including applications in biology, genetic systems and many other fields. For example, fractional-order differential equations may translate complex high-order dynamics into a concise and accurate mathematical model, and therefore, they are frequently used by researchers to address problems related to the physical behavior of certain complex or nonlinear phenomena [59]. Collectively, studies of fractal and fractional dynamics in biological systems are included in fractal and fractional problems in biology and life science as a whole.

Considering gene expression as a biological phenomenon inherent in all living cells, one can see that this process is tightly controlled, allowing the cell to respond to environmental changes. Gene expression is a stable, strictly hierarchically organized network of interaction between elements and signaling pathways involved in the process of gene regulation in the cell. A large number of interacting biochemical reactions exhibit new cell properties such as homeostasis and resistance to disturbances [60]. The consistency of different signaling pathways within and between several cell types is the basis for many biological processes, such as tissue growth and repair [61]. Interactions can occur at many hierarchical levels, and signaling proteins can influence the regulation of the gene network resulting in a complex behavior [62]. Thus, the expression level of a number of genes is a consequence of the response to previous events with the corresponding induction. Nonlinear dynamics in gene regulation provides resistance to mutational changes and also allows for phenotypic variations in gene expression levels [63]. In the present study, its own characteristic profile of nonlinear signals corresponding to the gene expression in each breed was determined (Figure 2 and Figure 3), which is characterized by a different rise rate (i.e., *K* coefficient that reflects the slope of the graphical function) and the initial growth point (variable *a*).

The rank distribution of gene expression and the slopes of the respective regression curves that we used is well substantiated in theoretical and experimental studies. For example, in ecology, the rank distribution parameter is specific to the type of community and a certain ecosystem and is an indicator of the community wellbeing. A generally accepted indicator of the state of an ecosystem is a variety of diversity indices, which are associated with the parameters of rank distributions [64]. There are different explanations for the shape of the rank distributions. In particular, according to Motomura’s exponential law, the abundance of a species is explained by its success in competition with other species for the limited resource available in the ecosystem [65]. One of the mandatory requirements for variables in regression analysis is that the independent variable must be a deterministic and continuous value, which is not observed for discrete ranks, but this principle is still employed in ecology [66]. The fractality property of the species structure organization in a biotic community is a generally accepted fact [4,5]. Based on the idea of self-similarity of systems, ranging from the organization of a species community to the regulation of biochemical processes in the body, we have applied here some approaches typical for the analysis of fractals. The use of the slope on graphs of various dependencies has also become part of the biological research practice. Yakimov [12] summarized that “as a result, the slope of the exponential dependence *z* has become so entrenched in the minds of ecologists as a standard characteristic of the spatial structure of communities that it is often the subject of statistical meta-analysis [67].” In the course of multifractal analysis, Yakimov [12] assessed the spectrum of indicators, for which he used the slopes of the linear regression for dependences of moments on the scale in logarithmic coordinates. Examples of similar studies are also known (e.g., [68]), in which the exponential dependence is presented in log–log coordinates.

Furthermore, one could interpret the resulting MGEI by answering the question of what it means in different breeds from a physiological point of view, i.e., in terms of characterization of muscle development (breast and thigh) in chicks from a day-old to 4-weeks-old. According to the data for relationships between the coefficients and traits (Figure 3), we have identified a significant correlation between the following indicators: on the one hand, *K*(br) and MGEI in E14 embryos and, on the other, body weight (as well as GR) in 2- and 4-week-old chicks. Thus, we can suggest that the coefficients *K*(br) and MGEI in embryos can serve as predictors of future growth in hatched chicks. This may be the biological interpretation of these coefficients, but with the caveat that they can act as predictors within specific data and their transfer to other experimental data should be treated with a caution. It can also be said that the methodology applied here requires further confirmation on a larger sample of data.

The same trends obtained for MGEI can be observed for MGEFDI derived using fractal dimension coefficients *D*(br) and *D*(th). In the present investigation, we noted a certain discrepancy in the network of genes in different muscle types and tried to level it through the introduction of MGEI and, then, MGEFDI. In further research, with the aim of non-manual selection of gene ranks in each breed/muscle type, it would be desirable to develop an algorithm to find the best *R*^2^ estimates and apply it to a machine search for optimal gene rank compositions. Since the amount of data in this study was rather small, we visually assessed the need to increase *R*^2^ and manually chose the most appropriate series of points (*N*) to determine *D*. Note that a different strategy was used for the *K* coefficients, and the respective models were characterized by fairly high *R*^2^ coefficients. Based on our findings, we can speculate that higher *D*(br) and *D*(th) values may indicate lesser artificial selection effects and greater natural selection effects during the domestic chicken evolution. All studied breeds are characterized by their own fractal dimension profiles with certain features, e.g., for meat breeds we observe lower *D*(br) values. Our analysis also showed that MGEFDI is not likely to be directly related to BW28 (Table S11), but it appeared to relate to breed utility type. The UG game breed has natural qualities necessary for survival, and therefore, it is characterized by high MGEFDI and MGEI indices. Broiler breeds, on the other hand, have unsuitable qualities for a natural survival, and therefore, their lower MGEFDI and MGEI values suggest that these breeds were more subject to artificial selection. In general, it can be assumed that the combined incorporation of the *K* and *D* coefficients from different muscle types into the respective integral indices MGEI and MGEFDI enables the characterization of the breed type to a greater extent than growth traits (see further discussion in SI S3).

When developing the second fractal model, we used the concept of the bioconsolidation index (*Ind*) that varies within 0…1. We believe that if *Ind* is equal to one, gene expressions are strongly matched in their intensity and correspond to the fractal concept. If *Ind* is zero, the expressions are completely mismatched and did not conform to the fractal concept at all. In reality, the index acquires intermediate values. We believe that if the conditions for the development of chickens, their feeding and keeping are optimal for the implementation of concordant gene expressions, *Ind* will tend to higher values. Consequently, we suggest that *Ind* enables to monitor the state of biochemical and physiological processes inside the animal body using such genetic indicators as gene expressions.

Thus, our findings suggest that different breeds are initially programmed for a different balance of myogenesis gene expression. A detailed analysis of the gene expression network can help speed up the selection progress toward improved poultry performance. The lower correlation of the bioconsolidation index with egg weight quite expectedly indicates a relatively weak relationship between the expressions of the considered myogenesis genes and egg traits. The high correlation of the bioconsolidation index with the body weight of chicks over the entire observation period may be indicative of a high consistency of the myogenesis gene expression processes in the embryonic period with the growth of chicks in the initial growing period post hatch. On the other hand, the higher correlation of embryonic NO metabolism with the body weight of chicks at a day-old and the subsequent decline of this correlation may be related to the attenuation of the corresponding biochemical processes of NO oxidation as the chicks develop.

## 5. Concluding Remarks

One of the diagnostic features of a fractal object is the power dependence of its considered elements [18]. Using a fractal approach, we developed here two mathematical models to search for such relationships in the expression of seven embryonic myogenesis genes in chickens and found patterns of their conformity. At the same time, significant differences were found in the fractal properties of myogenesis genes in two types of muscle tissue, breast and thigh, and in embryos of different breeds that represent four main types of chicken biodiversity and utility (i.e., meat, dual purpose, egg and game).

In the framework of the first model, it was proposed for each breed to use the slope of the regression dependences of expression on the rank gradation of genes (*K*) and the fractal dimensions of expression (*D*). In particular, regression curves were plotted that describe fractal patterns of gene expression in each breed, taking into account the rank gradation of genes. To summarize the coordinated network of myogenesis genes in the breast and thigh muscles of chick embryos, two integral indices were proposed: MGEI as the ratio of the *K* coefficients in two types of muscles and MGEFDI as the ratio of the *D* coefficients. Following the results of fractal and correlation analyzes and based on the features of the coordinated network of genes in birds of different breeds, a specific, convincing model was generated that was described by regression curves and linked such physiological (phenotypic) indicators as GR and productivity with genetic features. In general, this model and the indices used adequately reflect the fractal patterns of myogenesis gene expression, show a correlation with the postnatal GR and, importantly, fit into the established system of breed diversity in chickens. The second fractal model is based on deriving breed-specific fractal portraits and bioconsolidation indices of gene expression that provide an additional characteristic of genes’ interplay. Bioconsolidation indices characterize the features of the location of genes in fractal portraits among the considered chicken breeds. The presence of increased efficiency of biochemical and genetic processes in the chicken body at a higher level of fractal bioconsolidation of myogenesis gene expression has been suggested. In addition, a possible relationship between the gene expression bioconsolidation indices in the embryo muscles and the levels of NO metabolites and GR of chicks was discovered.

Overall, the fractal approaches and models helped in this study to expand our understanding of the coordinated network of embryonic myogenesis genes and their role in early development and growth, as well as implement additional characteristics of genetic diversity in chickens. Thus, the novel findings from this study imply the complex and coordinated work of myogenesis genes in the embryogenesis stages, which also reflects breed-specific and unique patterns of these processes. The present investigation reveals definite differences in the early formation of genetic potentials and subsequent manifestation of utility types in divergently selected breeds. The further practical significance of the present research lies in the fact that the applied methods, models and metrics can characterize the degree of genetic variability of the meat-type and other breeds created using the respective divergent targets of artificial selection. In addition, the approaches developed and tested here can be instrumental in assessing how strong the impact of divergent selection is in particular breeds. The obtained data can be of greater practical interest for breeders and geneticists who work with divergently selected poultry breeds.

## Figures and Tables

**Table 1:** List of the studied chicken breeds.

Breed	Code	Type of Breed Utility ^1^	Origin
Broiler	BR	Meat	Russia
White Cornish	WC	Meat	England/Russia
Plymouth Rock White	PRW	Dual purpose (meat-egg type)	USA/Russia
Yurlov Crower	YC	Dual purpose (meat-egg type)	Russia
Brahma Buff	BB	Dual purpose (egg-meat type)	USA/India
Orloff Mille Fleur	OMF	Dual purpose (meat-egg type)	Russia
Layer	LR	Egg	The Netherlands
Uzbek Game (or Kulangi)	UG	Game	Uzbekistan

^1^ Chicken breed types according to the phenotypic clustering model [13].

**Table 2:** Relative gene expression levels defined by FC values in the breast and thigh muscles of E14 chick embryos as estimated in the studied breeds.

Muscles	Genes *	Breeds							
		Broiler	White Cornish	Plymouth Rock White	Yurlov Crower	Brahma Buff	Orloff Mille Fleur	Layer	Uzbek Game
Breast	*MSTN*	11.55	4.89	6.59	121.9	41.07	2.41	4.72	1.18
	*GHR*	6.63	5.62	4.35	69.1	31.78	3.32	4.79	2.51
	*MEF2C*	6.59	2.91	4	302.3	219.8	2.33	4.14	1.45
	*MYOD1*	11.31	2.19	2.87	−7.11	−25.46	16.11	4.59	−81.01
	*MYOG*	7.46	−4.32	78.25	2.04	−1.95	5.58	1.03	−106.9
	*MYH1*	−41,760.00	−16.22	−24.42	1.07	−1.73	−16,270.00	−29.45	−11,990.00
	*MYF5*	−7.57	−685.02	−4.76	−5.90	−8.57	−37.53	−66.26	−4.47
Thigh	*MSTN*	3.86	4.03	4.5	46.53	8.86	−1.28	1.25	3.25
	*GHR*	3.07	3.05	2.95	26.1	3.72	1.62	1.31	4.92
	*MEF2C*	2.36	−1.69	−1.02	494.56	63.39	1.78	2.46	−4.79
	*MYOD1*	18.77	−12.13	13.18	28.44	8.78	6.06	1.08	−13.93
	*MYOG*	6.73	−4640.29	1.39	1.39	−2.70	3.63	−78.25	−118.60
	*MYH1*	−10,020.00	−335.46	−115.36	2.3	1.37	−8481.00	6.23	−17,560.00
	*MYF5*	−6.45	−25,531.63	−33.36	195.36	38.02	−18.90	−87.43	−2.43

* *MSTN*, myostatin; *GHR*, growth hormone receptor; *MEF2C*, myocyte enhancer factor 2C; *MYOD1*, myogenic differentiation 1; *MYH1*, myosin heavy chain 1; *MYOG*, myogenin; *MYF5*, myogenic factor 5.

**Table 3:** Breed scores for phenotypic/metabolic traits and *K* * coefficients in the breast (*K*(br)) and thigh (*K*(th)) muscles.

Breed	Body Weight, g			Growth Rate		E7 Levels of NO Metabolites						Breed Type Category (ANOVA, *p* < 0.05)	*K*(br)	*K*(th)	MGEI	FC (*GHR*)
	1 Day	14 Days	28 Days	2 Weeks	4 Weeks	NOD, µM	NOD Category (ANOVA, *p* > 0.05)	Nitrate, µM	Nitrate Category (ANOVA, *p* > 0.05)	NO Oxidation, %	NO Oxidation Category (ANOVA, *p* > 0.05)					
Broiler	47.5	305.0	1157.0	6.42	24.36	3.3	Low	145.4	High	98.1	High	Meat	0.012	0.082	0.147	6.63
White Cornish	49.3	291.7	1287.5	5.92	26.12	9.5	Low	152.2	High	96.9	High	Meat	0.037	0.233	0.159	5.62
Plymouth Rock White	44.9	265.2	1058.4	5.91	23.57	141.8	High	0	Low	2.6	Low	Meat	0.061	0.072	0.853	4.35
Yurlov Crower	39.0	101.3	240.0	2.60	6.15	149.6	High	0	Low	2.0	Low	No	0.316	0.584	0.541	69.1
Brahma Buff	38.2	107.5	241.7	2.81	6.33	36.0	Low	100.0	High	74.1	High	No	0.119	0.151	0.788	31.78
Orloff Mille Fleur	35.5	93.1	167.8	2.62	4.73	131.5	High	0	Low	2.1	Low	No	0.096	0.057	1.684	3.32
Layer	42.4	79.8	222.4	1.88	5.25	138.9	High	0	Low	2.4	Low	No	0.070	0.026	2.735	4.79
Uzbek Game	41.3	92.4	223.7	2.24	5.42	8.8	Low	143.4	High	96.9	High	No	0.300	0.061	4.905	2.51

* Coefficients (slope angles) *K* from the respective regression equations (Figure 2). Abbreviations: Nitrate, content of nitrate; NOD, nitric oxide donors; ANOVA, analysis of variance; NO oxidation, rate of NO oxidation to nitrate; MGEI = *K*(br)/*K*(th), myogenesis gene expression index; FC, fold change.

**Table 4:** Estimation of fractal dimension coefficients *D* in the breast (*D*(br)) and thigh (*D*(th)) muscles.

Breed	Breast		Thigh		MGEFDI **
	*D*(br)	*N* *	*D*(th)	*N*	
Broiler	0.400	[1:6]	0.954	[1:4]	0.419
White Cornish	1.289	[1:6]	11.211	[3:7]	0.115
Plymouth Rock White	0.717	[1:4]	1.509	[1:4]	0.475
Yurlov Crower	4.104	[1:6]	3.377	[1:4]	1.215
Brahma Buff	2.968	[2:7]	2.122	[1:4]	1.399
Orloff Mille Fleur	2.984	[2:6]	1.1325	[1:4]	2.634
Layer	3.109	[1:6]	1.859	[1:4]	1.673
Uzbek Game	3.740	[1:6]	0.632	[1:4]	5.915

* *N*, range of gene ranks specifically chosen for each breed/muscle type; ** MGEFDI = *D*(br)/*D*(th), myogenesis gene expression fractal dimension index.

## Data Availability

The original contributions presented in the study are included in the article and Supplementary Materials, further inquiries can be directed to the corresponding author.

## Supplementary Materials

The following supporting information can be downloaded at: https://www.mdpi.com/article/10.3390/ani13030521/s1, Supplementary Information S1: Methodological substantiation for assessing the NO oxidation rate as described in refs [27,47,48,69,70,71,72,73,74]; Supplementary Information S2: Search for the relationship between fractal dimension coefficients D and body weight of birds; Supplementary Information S3: Extended discussion on the developed mathematical models and indices; Figure S1: Log–log plots of |FC − 1|(N) dependences for gene expression data (Table 2) in the breeds by muscle type. (A) Breast muscles; (B) thigh muscles; Figure S2: Special cases of |FC − 1|(*N*) regressions for different breeds by muscle groups; Figure S3: Estimation of fractal dimension coefficients *D*; Figure S4: Fractal portraits of gene expression in the breast (A1–G1) and thigh (A2–G2) muscles of embryos in the chicken breeds studied; Table S1: Rank distribution of the considered breast muscle genes according to the increasing level of their differential expression; Table S2: Rank distribution of the considered thigh muscle genes according to the increasing level of their differential expression (after |FC − 1| transformation); Table S3: Ordered (by descending) breed-specific values of the *K*(br) coefficient in thigh muscles compared to body weight of chicks at 14 and 28 days; Table S4: Relationships between *K* coefficients and phenotypic/metabolic traits when comparing Pearson and Spearman correlations; Table S5: Indicators *K*(br), *K*(th) and *K*(br)/*K*(th) depending on the utility type of breeds; Table S6: Coefficients and FC values of the *GHR* gene when sorted by MGEI* and depending on the utility type of breeds; Table S7: Dynamics of changes in the body weight of chicks over a 4-week period; Table S8: Dynamics of changes in body weight and growth rate of chicks over 2- and 4-week periods in comparison with the FC values of the *GHR* gene; Table S9: Spearman correlation coefficient values for 11 indicators of growth, NO metabolism and myogenesis across all the breeds studied; Table S10: Spearman correlation *p*-values for 11 indicators shown in Supplementary Table S9; Table S11: Summary table for comparing coefficients *D*, *K*, *MGEFDI* and *MGEI*; Table S12: Expression levels of myogenesis genes (fold change values) in the tissues of the breast and thigh muscles of E14 chick embryos on the example of the WC breed; Table S13: Transformation of numerical indicators of fold change (FC) for gene expression in the breast muscles of WC chick embryos into Y- and X-coordinates of points representing genes in a fractal portrait; Table S14: Correlation of the bioconsolidation index with the phenotypic characteristics of embryos and chicks of various chicken breeds.

## References

[1] Morse D.R., Lawton J.H., Dodson M.M., Williamson M.H. (1985). Fractal dimension of vegetation and the distribution of arthropod body lengths. Nature.

[2] Şişli A.B., Pahnvar A.J., Engin M., Engin E.Z. (2020). Obtaining the heart rate information from the speckle images by fractal analysis method. Celal Bayar Üniv. Fen Bilim. Derg..

[3] Lee C.Y. (2020). The fractal dimension as a measure for characterizing genetic variation of the human genome. Comput. Biol. Chem..

[4] Yakimov B.N., Solntsev L.A., Rozenberg G.S., Iudin D.I., Gelashvili D.B. (2014). Scale invariance of biosystems: From embryo to community. Russ. J. Dev. Biol..

[5] Yakimov B.N., Solntsev L.A., Rozenberg G.S., Iudin D.I., Shirokov A.I., Lokteva O.A., Gelashvili D.B. (2014). Local multifractal analysis of the spatial structure of meadow comminities at small scale. Dokl. Biol. Sci..

[6] Falconer K. (2003). Fractal Geometry.

[7] Rihan F.A. (2013). Numerical modeling of fractional-order biological systems. Abstr. Appl. Anal..

[8] Pecl G.T., Araújo M.B., Bell J.D., Blanchard J., Bonebrake T.C., Chen I.C., Clark T.D., Colwell R.K., Danielsen F., Evengård B. (2017). Biodiversity redistribution under climate change: Impacts on ecosystems and human well-being. Science.

[9] Lewin H.A., Robinson G.E., Kress W.J., Baker W.J., Coddington J., Crandall K.A., Durbin R., Edwards S.V., Forest F., Gilbert M.T.P. (2018). Earth BioGenome Project: Sequencing life for the future of life. Proc. Natl. Acad. Sci. USA.

[10] Zhang M., Peng W.F., Hu X.J., Zhao Y.X., Lv F.H., Yang J. (2018). Global genomic diversity and conservation priorities for domestic animals are associated with the economies of their regions of origin. Sci. Rep..

[11] Yang G., Ryo M., Roy J., Hempel S., Rillig M.C. (2021). Plant and soil biodiversity have non-substitutable stabilising effects on biomass production. Ecol. Lett..

[12] Yakimov V.N. (2015). Methodology for the Analysis of Scaling Taxonomic, Phylogenetic and Functional Diversity of Biotic Communities. D.Sc. Thesis.

[13] Larkina T.A., Barkova O.Y., Peglivanyan G.K., Mitrofanova O.V., Dementieva N.V., Stanishevskaya O.I., Vakhrameev A.B., Makarova A.V., Shcherbakov Y.S., Pozovnikova M.V. (2021). Evolutionary subdivision of domestic chickens: Implications for local breeds as assessed by phenotype and genotype in comparison to commercial and fancy breeds. Agriculture.

[14] Ryabokon Y.O., Mykytyuk D.M., Frolov V.V., Katerynych O.O., Bondarenko Y.V., Mosyakina T.V., Gadyuchko O.T., Kovalenko G.T., Bogatyr V.P., Lyuty Y.S., Ryabokon Y.O. (2005). Catalog of Poultry Breeding Resources of Ukraine.

[15] Khvostyk V.P., Bondarenko Y.V. (2016). Hereditary load in chicken populations of the domestic gene pool. Visnyk Sumsʹkoho Natsionalʹnoho Ahrarnoho Universytetu [Bull. Sumy Natl. Agrar. Univ.].

[16] Khvostyk V.P., Bondarenko Y.V. (2017). Informational and statistical parameters of body weight of chickens of the domestic gene pool. Visnyk Sumsʹkoho Natsionalʹnoho Ahrarnoho Universytetu Seriya Tvarynnytstvo [Bull. Sumy Natl. Agrar. Univ. Ser. Livest.].

[17] Tagirov M.T., Tereshchenko L.V., Tereshchenko A.V. (2006). Substantiation of the possibility of using primary germ cells as material for the preservation of poultry genetic resources. Ptakhivnytstvo.

[18] Feder J. (1988). Fractals.

[19] Morozov A.D. (2002). Introduction to the Theory of Fractals.

[20] Schroeder M.R. (1991). Fractals, Chaos, Power Laws. Minutes from an Infinite Universe.

[21] MacArthur R.H., Wilson E.O. (1963). An equilibrium theory of insular zoogeography. Evolution.

[22] Vorob’ev N.I., Nikonov I.N., Selina M.V. (2020). Mathematical model of determining the index of fractal structures for estimating efficiency of probiotic fodder additives for microbiotes of the gut intestin. Vet. Zootekhniya I Biotekhnologiya [Vet. Med. Zootech. Biotechnol.].

[23] Kochish I.I., Vorobyov N.I., Nikonov I.N., Selina M.V. Fractal Bioconsolidation of Microorganisms in the Intestines of Laying Hens Due to the Use of a Feed Additive from the Mineral Shungite. Proceedings of the Materials of the 2nd International Scientific and Practical Conference on Molecular Genetic Technologies for Analysis of Gene Expression Related to Animal Productivity and Disease Resistance.

[24] Titov V.Y., Dolgorukova A.M., Fisinin V.I., Borkhunova E.N., Kondratov G.V., Slesarenko N.A., Kochish I.I. (2018). The role of nitric oxide (NO) in the body growth rate of birds. Worlds Poult. Sci. J..

[25] Titov V.Y., Kochish I.I., Nikonov I.N., Korenyuga M.V., Myasnikova O.V., Kuvanov T.K., Dolgorukova A.M. Genetic Markers of Meat Performance in Poultry. Proceedings of the Materials of the 2nd International Scientific and Practical Conference on Molecular Genetic Technologies for Analysis of Gene Expression Related to Animal Productivity and Disease Resistance.

[26] Titov V., Dolgorukova A., Khasanova L., Kochish I., Korenyuga M. (2021). Nitric oxide (NO) and arginine as factors for increasing poultry meat productivity. KnE Life Sci..

[27] Titov V.Y., Dolgorukova A.M., Kochish I.I. What Gene Expression is Associated with Nitric Oxide Oxidation in the Avian Embryo?. Proceedings of the Materials of the 3rd International Scientific and Practical Conference on Molecular Genetic Technologies for Analysis of Gene Expression Related to Animal Productivity and Disease Resistance.

[28] Cazzato D., Assi E., Moscheni C., Brunelli S., De Palma C., Cervia D., Perrotta C., Clementi E. (2014). Nitric oxide drives embryonic myogenesis in chicken through the upregulation of myogenic differentiation factors. Exp. Cell Res..

[29] Dolgorukova A.M., Titov V.Y., Kochish I.I., Fisinin V.I., Nikonov I.N., Kosenko O.V., Myasnikova O.V. (2020). The embryonic metabolism of nitric oxide and its interrelation with postembryonic development in chicken (*Gallus gallus domesticus* L.) and quails (*Coturnix coturnix* L.). Sel’skokhozyaistvennaya Biol. Agric. Biol..

[30] Ulibarri J.A., Mozdziak P.E., Schultz E., Cook C., Best T.M. (1999). Nitric oxide donors, sodium nitroprusside and S-nitroso-N-acetylpencillamine, stimulate myoblast proliferation in vitro. Vitr. Cell Dev. Biol. Anim..

[31] Anderson J.E. (2000). A role for nitric oxide in muscle repair: Nitric oxide-mediated activation of muscle satellite cells. Mol. Biol. Cell..

[32] Stamler J.S., Meissner G. (2001). Physiology of nitric oxide in skeletal muscle. Physiol. Rev..

[33] Long J.H., Lira V.A., Soltow Q.A., Betters J.L., Sellman J.E., Criswell D.S. (2006). Arginine supplementation induces myoblast fusion via augmentation of nitric oxide production. J. Muscle Res. Cell Motil..

[34] Li Y., Wang Y., Willems E., Willemsen H., Franssens L., Buyse J., Decuypere E., Everaert N. (2016). In ovo L-arginine supplementation stimulates myoblast differentiation but negatively affects muscle development of broiler chicken after hatching. J. Anim. Physiol. Anim. Nutr..

[35] Tirone M., Conti V., Manenti F., Nicolosi P.A., D’Orlando C., Azzoni E., Brunelli S. (2016). Nitric oxide donor molsidomine positively modulates myogenic differentiation of embryonic endothelial progenitors. PLoS ONE.

[36] Reddish J.M., Wick M., St-Pierre N.R., Lilburn M.S. (2005). Analysis of myosin isoform transitions during growth and development in diverse chicken genotypes. Poult. Sci..

[37] Tereshchenko O.V., Katerinich O.O., Pankova S.M., Borodai V.P. (2015). Formation of genetic resources of domestic breeds of poultry in the context of food security of the state. Sučasne Ptahìvnictvo.

[38] Bondarenko Y.V., Podstreshny A.P. (1996). Genetic Monitoring of Chicken Populations. Abstracts of the 2nd International Conference on Molecular Genetic Markers of Animals.

[39] Romanov M.N., Weigend S., Bondarenko Y.V., Podstreshny A.P., Kutnyuk P.I., Sakhatsky N.I. Studies on Poultry Germplasm Diversity and Conservation in Ukraine. Proceedings of the Poultry Genetics Symposium.

[40] Tixier-Boichard M., Coquerelle G., Vilela-Lamego C., Weigend S., Barre-Dirrie A., Groenen M., Crooijmans R., Vignal A., Hillel J., Freidlin P. Contribution of Data on History, Management and Phenotype to the Description of the Diversity between Chicken Populations Sampled within the AVIANDIV Project. Proceedings of the Poultry Genetics Symposium.

[41] Tkachik T.E., Kutnyuk P.I., Bondarenko Y.V. (2005). Genetic load in land poultry populations. Ptakhivnytstvo.

[42] Weigend S., Romanov M.N., Rath D. Methodologies to Identify, Evaluate and Conserve Poultry Genetic Resources. Proceedings of the XXII World’s Poultry Congress & Exhibition: Participant List & Full Text CD + Book of Abstracts.

[43] Narushin V.G., Laptev G.Y., Yildirim E.A., Ilina L.A., Filippova V.A., Kochish I.I., Gorfunkel E.P., Dubrovin A.V., Novikova N.I., Novikova O.B. (2020). Modelling effects of phytobiotic administration on coherent responses to Salmonella infection in laying hens. Ital. J. Anim. Sci..

[44] Vorobiev N.I., Kochish I.I., Titov V.Y., Nikonov I.N., Korenyuga M.V., Myasnikova O.V., Kuvanov T.K., Dolgorukova A.M. Dependence of the Dynamics of Chick Growth in Egg- and Meat-type Breeds on Fractal Bioconsolidation Index of Myogenesis Gene Expression. Proceedings of the Materials of the 3rd International Scientific and Practical Conference on Molecular Genetic Technologies for Analysis of Gene Expression Related to Animal Productivity and Disease Resistance.

[45] Livak K.J., Schmittgen T.D. (2001). Analysis of relative gene expression data using real-time quantitative PCR and the 2^−ΔΔCT^ method. Methods.

[46] Schmittgen T.D., Livak K.J. (2008). Analyzing real-time PCR data by the comparative *C*_T_ method. Nat. Protoc..

[47] Titov V.Y. (2011). The enzymatic technologies open new possibilities for studying nitric oxide (NO) metabolism in living systems. Curr. Enzym. Inhib..

[48] Titov V.Y., Kosenko O.V., Starkova E.S., Kondratov G.V., Borkhunova E.N., Petrov V.A., Osipov A.N. (2016). Enzymatic sensor detects some forms of nitric oxide donors undetectable by other methods in living tissues. Bull. Exp. Biol. Med..

[49] Leek J.T., Scharpf R.B., Bravo H.C., Simcha D., Langmead B., Johnson W.E., Geman D., Baggerly K., Irizarry R.A. (2010). Tackling the widespread and critical impact of batch effects in high-throughput data. Nat. Rev. Genet..

[50] RStudio Team RStudio Builds: 2022.07.1+554.

[51] RDocumentation lm: Fitting Linear Models. stats (version 3.6.2). RDocumentation. https://www.rdocumentation.org/packages/stats/versions/3.6.2/topics/lm.

[52] Wickham H. (2009). Ggplot2: Elegant Graphics for Data Analysis.

[53] Wickham H., Chang W., Henry L., Pedersen T.L., Takahashi K., Wilke C., Woo K., Yutani H., Dunnington D. (2021). ggplot2: Create Elegant Data Visualisations Using the Grammar of Graphics, Version 3.3.5.

[54] Pedersen T.L. (2021). ggplot2. Version 3.3.5. RDocumentation. https://www.rdocumentation.org/packages/ggplot2/versions/3.3.5.

[55] Wei T., Simko V. (2021). R Package ‘Corrplot’: Visualization of a Correlation Matrix. Version 0.90. https://github.com/taiyun/corrplot.

[56] Lv X., Zhang M., Li X., Ye R., Wang X. (2018). Transcriptome profiles reveal the crucial roles of auxin and cytokinin in the “shoot branching” of Cremastra appendiculata. Int. J. Mol. Sci..

[57] Yan J., Song J., Qiao M., Zhao X., Li R., Jiao J., Sun Q. (2019). Long noncoding RNA expression profile and functional analysis in psoriasis. Mol. Med. Rep..

[58] Sheluhin O.I., Magomedova D.I. (2017). Analysis of methods for calculating the fractal dimension of color and grayscale images. H&ES Res..

[59] Coman S., Boldisor C. (2022). Special Issue “Fractional Order Modeling in Interdisciplinary Applications” Fractal Fract., EISSN 2504–3110, Published by MDPI (Basel, Switzerland). https://www.mdpi.com/journal/fractalfract/special_issues/fractional_order_modeling.

[60] Ling H., Samarasinghe S., Kulasiri D. (2013). Novel recurrent neural network for modelling biological networks: Oscillatory p53 interaction dynamics. Biosystems.

[61] Zhao X.M., Li S. (2017). HISP: A hybrid intelligent approach for identifying directed signaling pathways. J. Mol. Cell. Biol..

[62] Bhalla U.S., Iyengar R. (1999). Emergent properties of networks of biological signaling pathways. Science.

[63] Steinacher A., Bates D.G., Akman O.E., Soyer O.S. (2016). Nonlinear dynamics in gene regulation promote robustness and evolvability of gene expression levels. PLoS ONE.

[64] Bulgakov N.G., Levich A.P. (2005). Description, origin and using of rank distribution in ecology of communities. Vestn. Mosk. Univ. Seriya 16 Biol..

[65] Motomura I. (1932). On the statistical treatment of assemblages. Zool. Mag..

[66] Bulgakov N.G., Levich A.P., Maksimov V.N., Gelashvili D.B. (2003). Regional ecological control based on biotic and abiotic monitoring data. Environmental Monitoring. Methods of Biological and Physico-Chemical Monitoring: Textbook.

[67] Drakare S., Lennon J.J., Hillebrand H. (2006). The imprint of the geographical, evolutionary and ecological context on species-area relationships. Ecol. Lett..

[68] Weron R. (2002). Estimating long-range dependence: Finite sample properties and confidence intervals. Physica A Stat. Mech. Appl..

[69] Vanin A.F. (2016). Dinitrosyl iron complexes with thiol-containing ligands as a “working form” of endogenous nitric oxide. Nitric Oxide.

[70] Vanin A.F., Borodulin R.R., Mikoyan V.D. (2017). Dinitrosyl iron complexes with natural thiol-containing ligands in aqueous solutions: Synthesis and some physico-chemical characteristics (A methodological review). Nitric Oxide.

[71] Tarpey M.M., Wink D.A., Grisham M.B. (2004). Methods for detection of reactive metabolites of oxygen and nitrogen: In vitro and in vivo considerations. Am. J. Physiol. Regul. Integr. Comp. Physiol..

[72] Titov V.Y., Osipov A.N. (2017). Nitrite and nitroso compounds can serve as specific catalase inhibitors. Redox Rep..

[73] Titov V.Y., Vinnikova E.Z., Akimova N.S., Fisinin V.I. (2012). Nitric oxide (NO) in bird embryogenesis: Physiological role and ability of practical use. Worlds Poult. Sci. J..

[74] Severina I.S., Bussygina O.G., Pyatakova N.V., Malenkova I.V., Vanin A.F. (2003). Activation of soluble guanylate cyclase by NO donors—S-nitrosothiols, and dinitrosyl-iron complexes with thiol-containing ligands. Nitric Oxide.

