# Supplementary material for: Features of Fractal Conformity and Bioconsolidation in the Early Myogenesis Gene Expression and Their Relationship to the Genetic Diversity of Chicken Breeds
Source: Animals (Basel). 2023 Feb 1;13(3):521. doi: 10.3390/ani13030521 (PMC9913260; doi:10.3390/ani13030521)
Supplement: Supplementary file 1 [file animals-13-00521-s001.zip › Supplementary Fig. S4.pdf]

Broiler

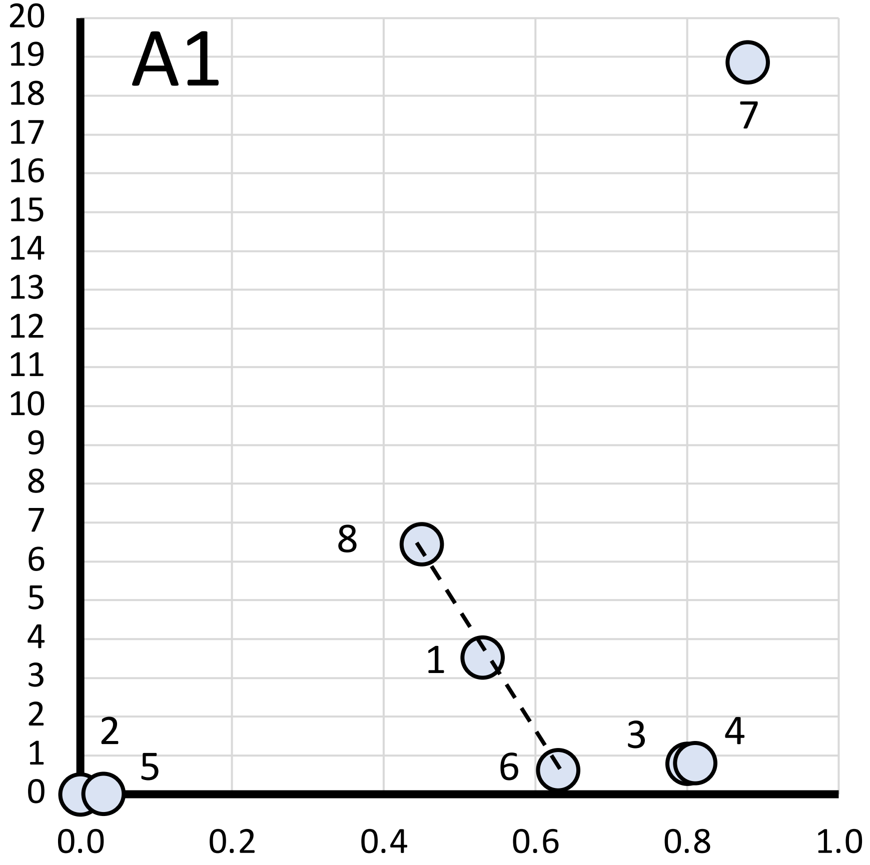

1	2	3	4	5	6	7	8
3.53	0	0.8	0.81	0.03	0.63	18.88	6.45
0.53	0	0.8	0.81	0.03	0.63	0.88	0.45

Breast muscles

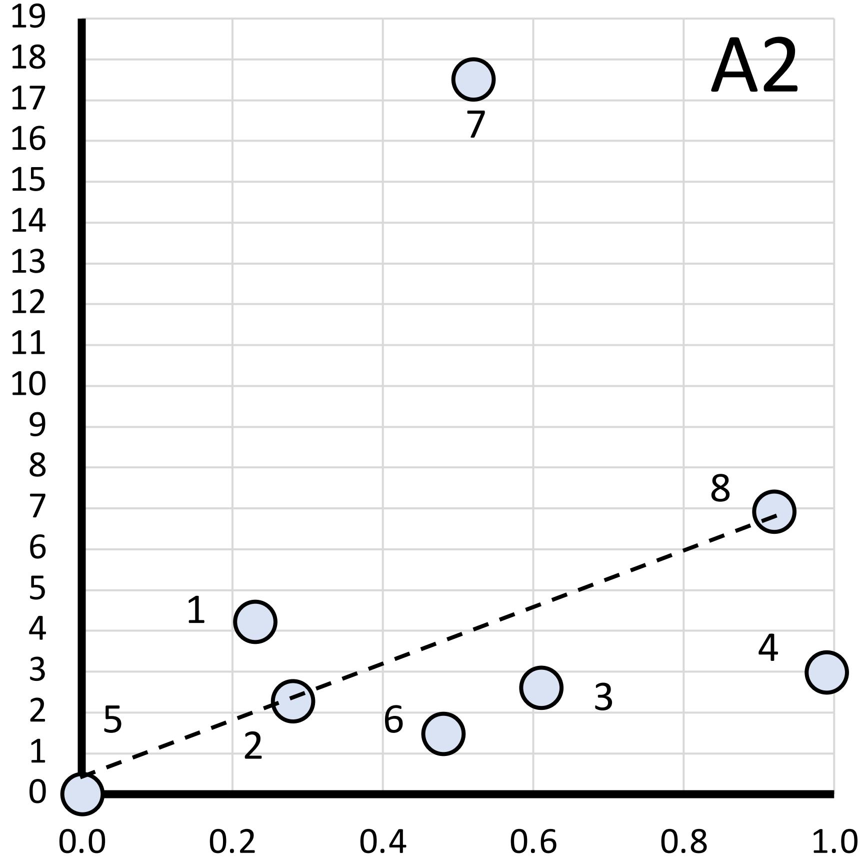

1	2	3	4	5	6	7	8
4.23	2.28	2.61	2.99	0	1.48	17.52	6.92
0.23	0.28	0.61	0.99	0	0.48	0.52	0.92

Thigh muscles

White Cornish

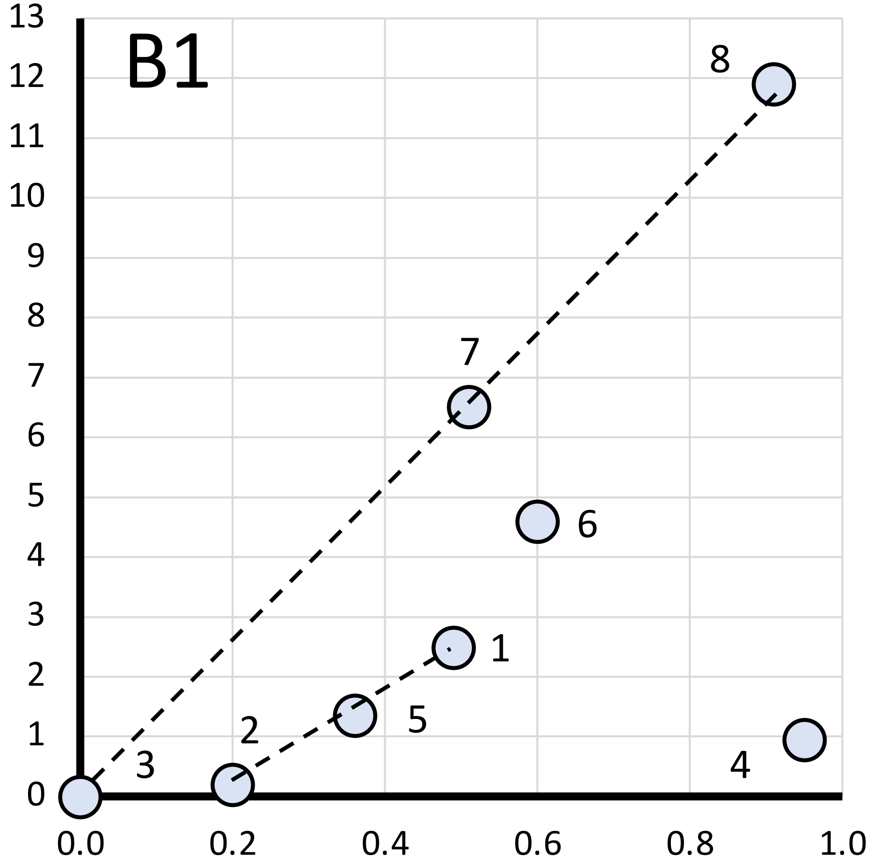

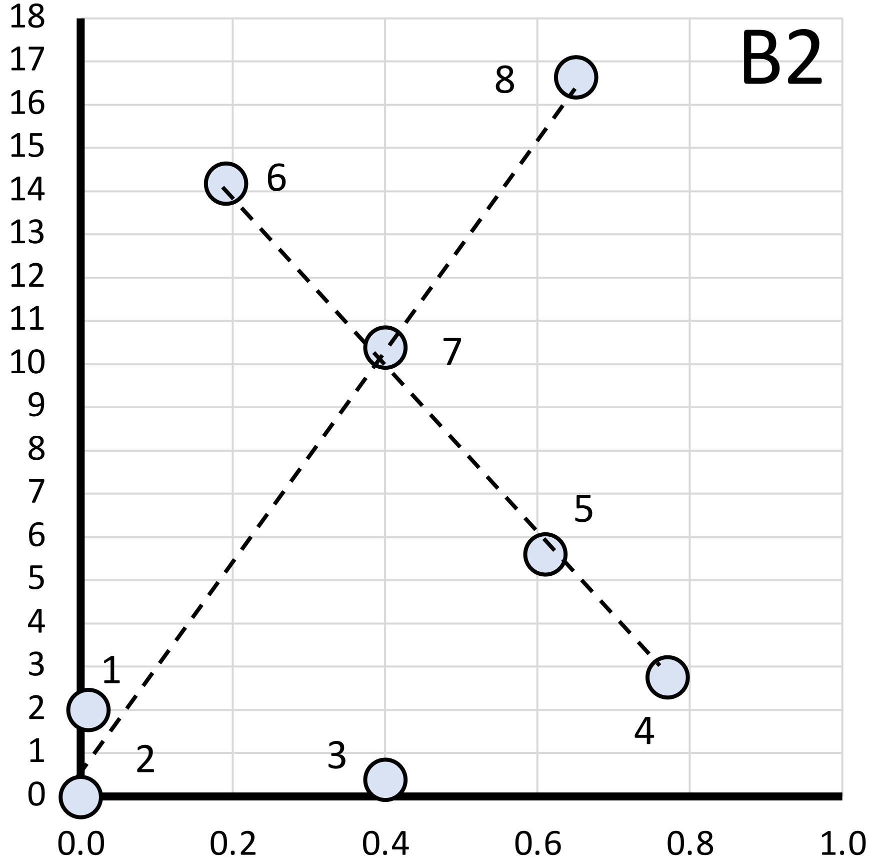

1	2	3	4	5	6	7	8
2.49	0.2	0	0.95	1.36	4.6	6.51	11.91
0.49	0.2	0	0.95	0.36	0.6	0.51	0.91

Breast muscles

1	2	3	4	5	6	7	8
2.01	0	0.4	2.77	5.61	14.19	10.4	16.65
0.01	0	0.4	0.77	0.61	0.19	0.4	0.65

Thigh muscles

Plymouth Rock White

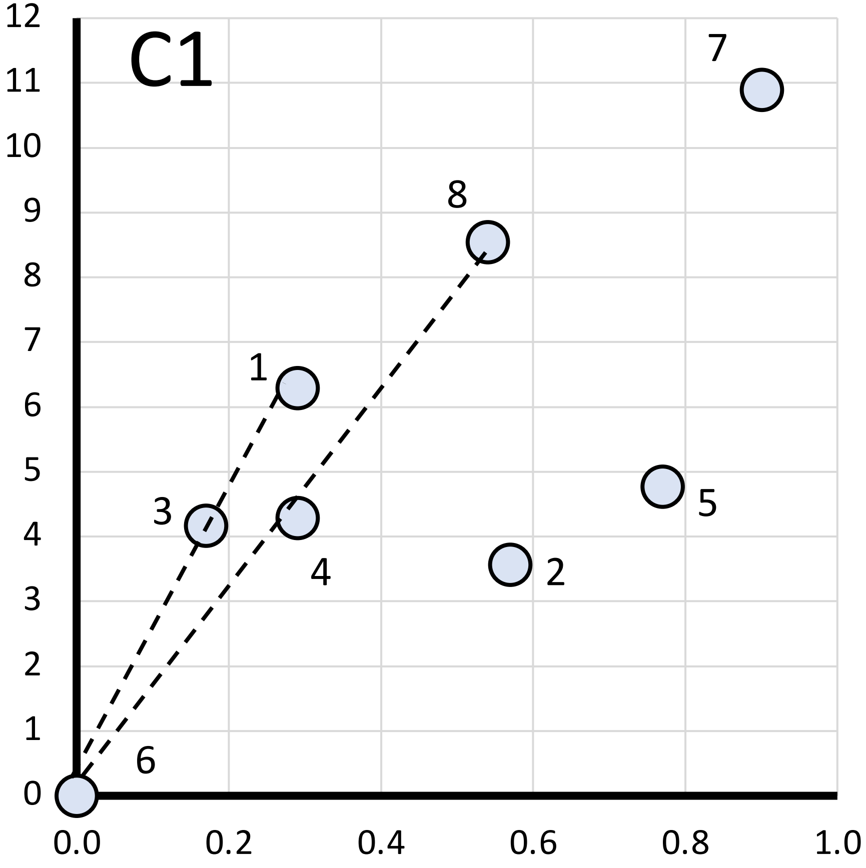

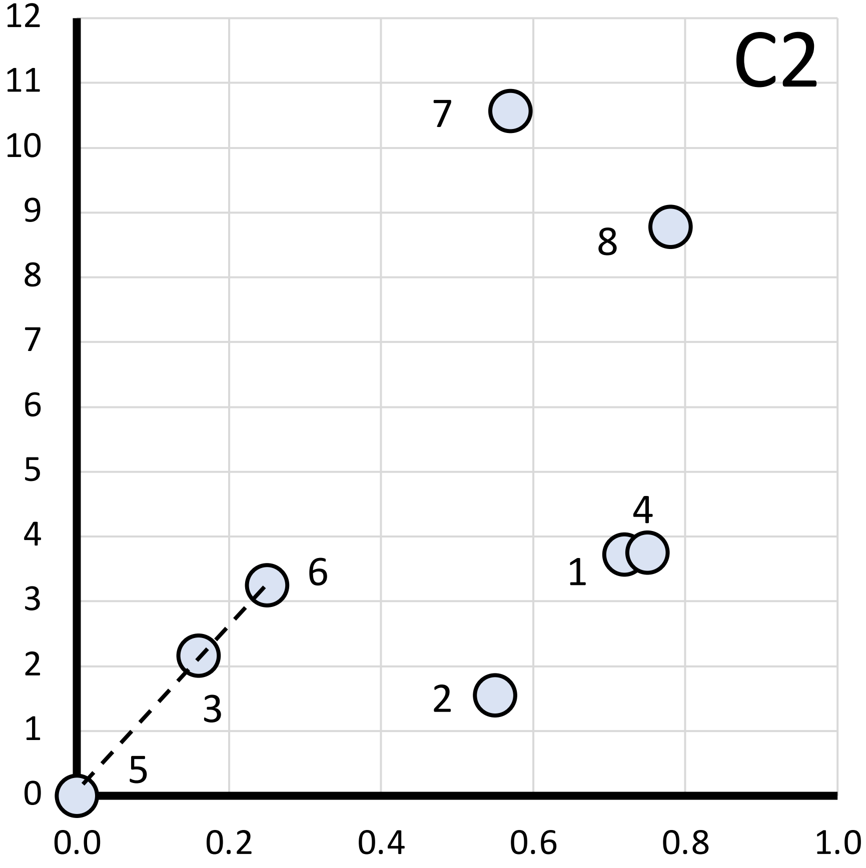

1	2	3	4	5	6	7	8
6.29	3.57	4.17	4.29	4.77	0	10.9	8.54
0.29	0.57	0.17	0.29	0.77	0	0.9	0.54

Breast muscles

1	2	3	4	5	6	7	8
3.72	1.55	2.16	3.75	0	3.25	10.57	8.78
0.72	0.55	0.16	0.75	0	0.25	0.57	0.78

Thigh muscles

Yurlov Crower

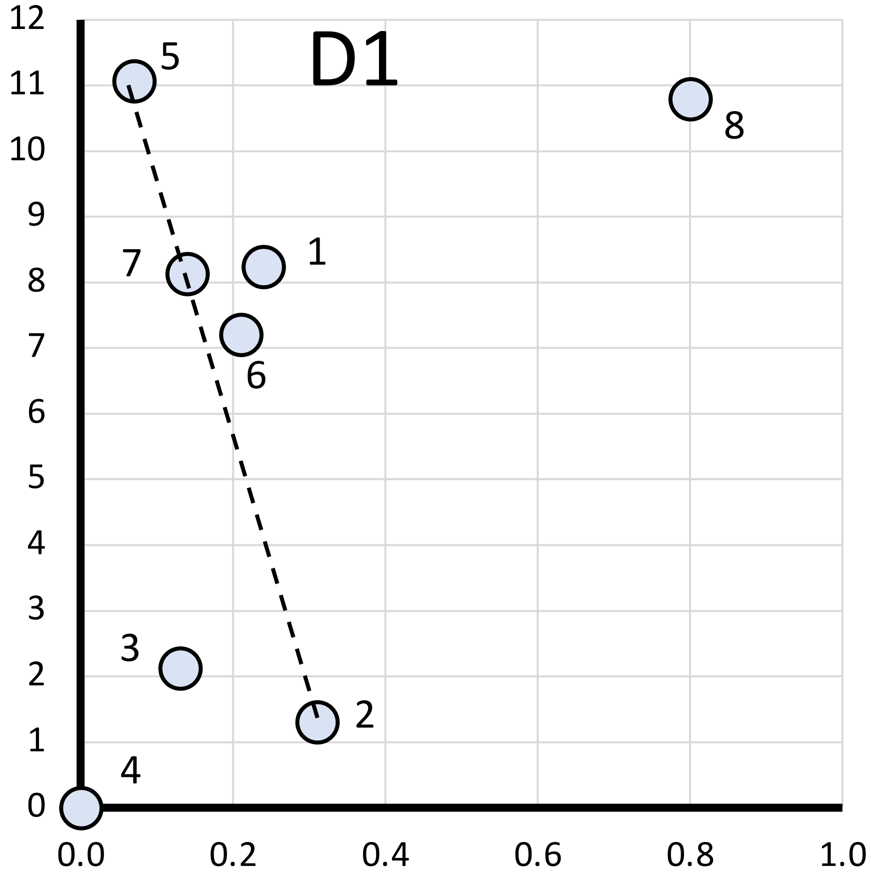

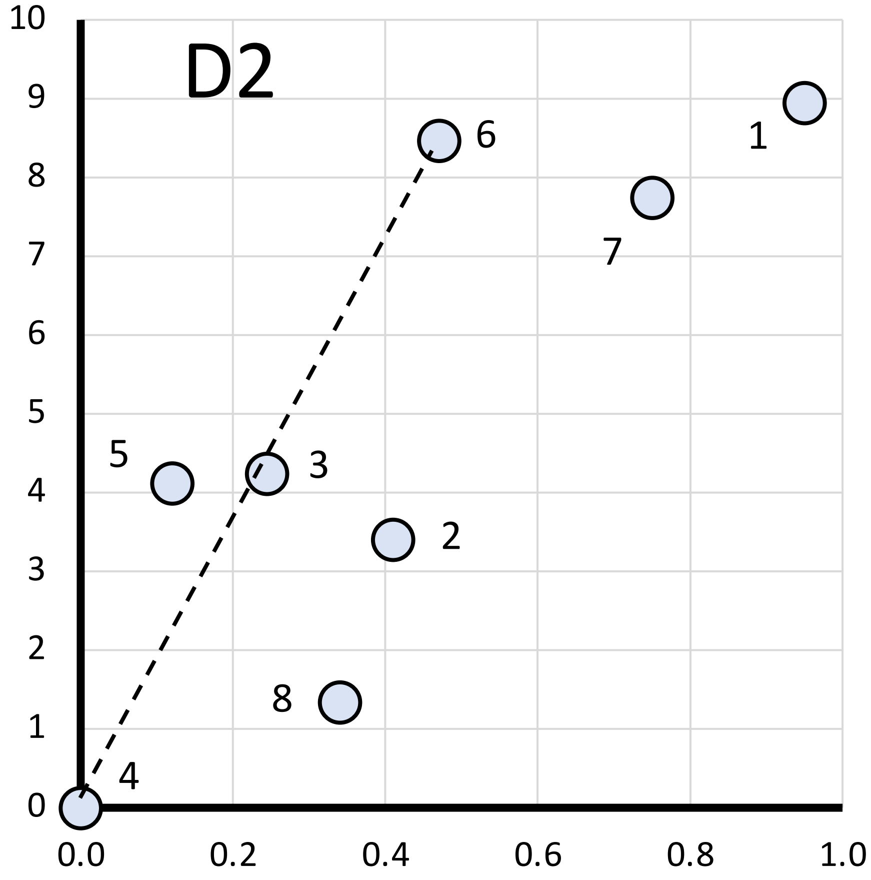

1	2	3	4	5	6	7	8
8.24	1.31	2.13	0	11.07	7.21	8.14	10.8
0.24	0.31	0.13	0	0.07	0.21	0.14	0.8

Breast muscles

1	2	3	4	5	6	7	8
8.95	3.41	4.244	0	4.12	8.47	7.75	1.34
0.95	0.41	0.244	0	0.12	0.47	0.75	0.34

Thigh muscles

Brahma Buff

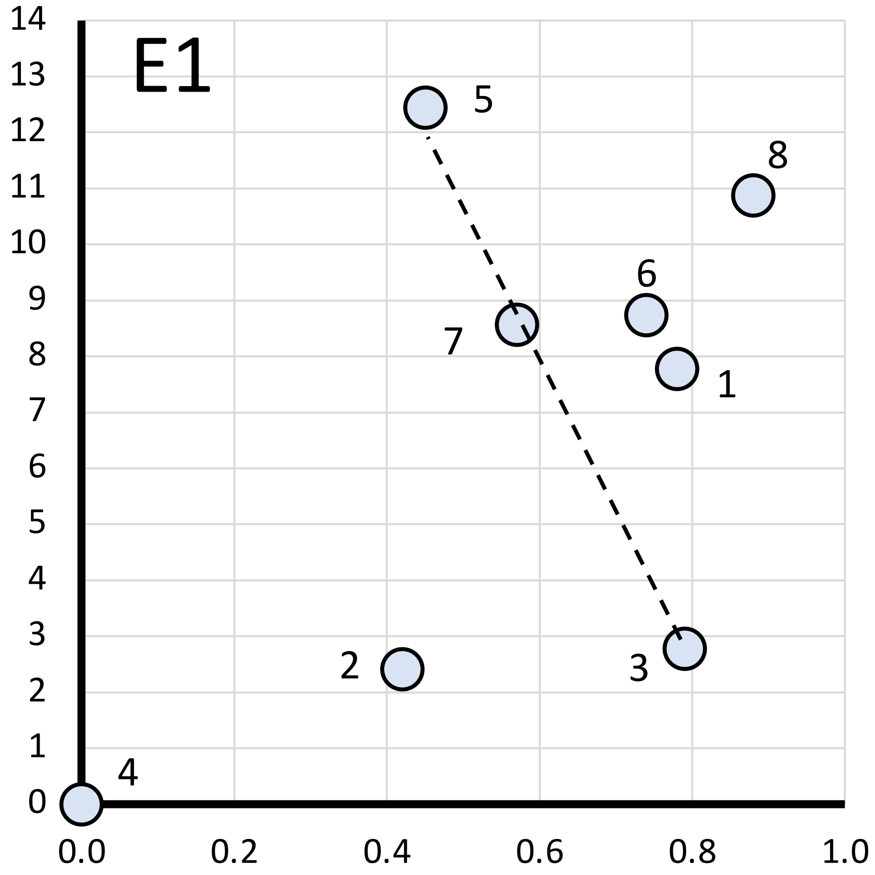

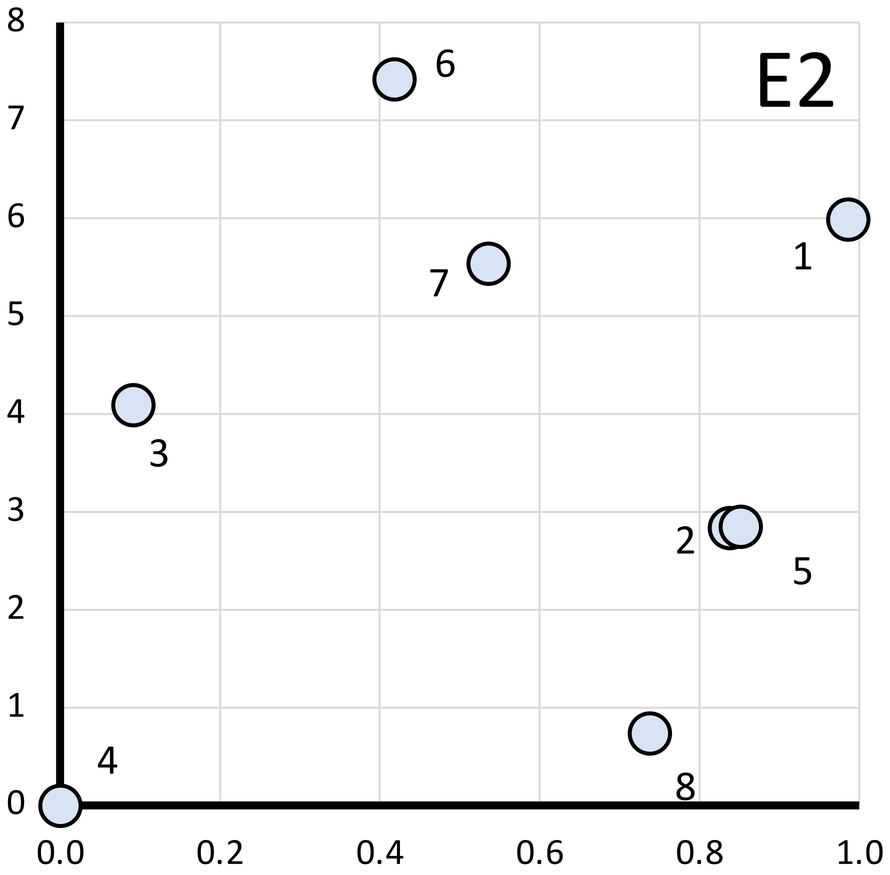

1	2	3	4	5	6	7	8
7.78	2.42	2.79	0	12.45	8.74	8.57	10.88
0.78	0.42	0.79	0	0.45	0.74	0.57	0.88

1	2	3	4	5	6	7	8
5.99	2.84	4.09	0.00	2.85	7.42	5.54	0.74
0.986295	0.838194	0.091117	0	0.851449	0.418272	0.536296	0.73779

Breast muscles

Thigh muscles

Orloff Mille Fleur

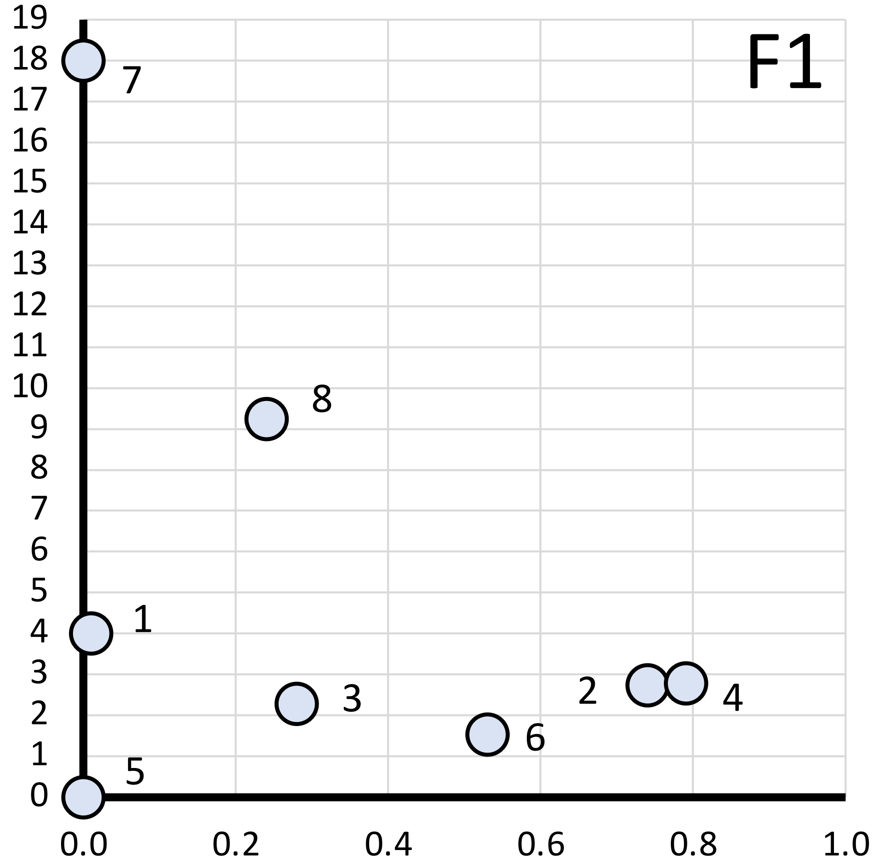

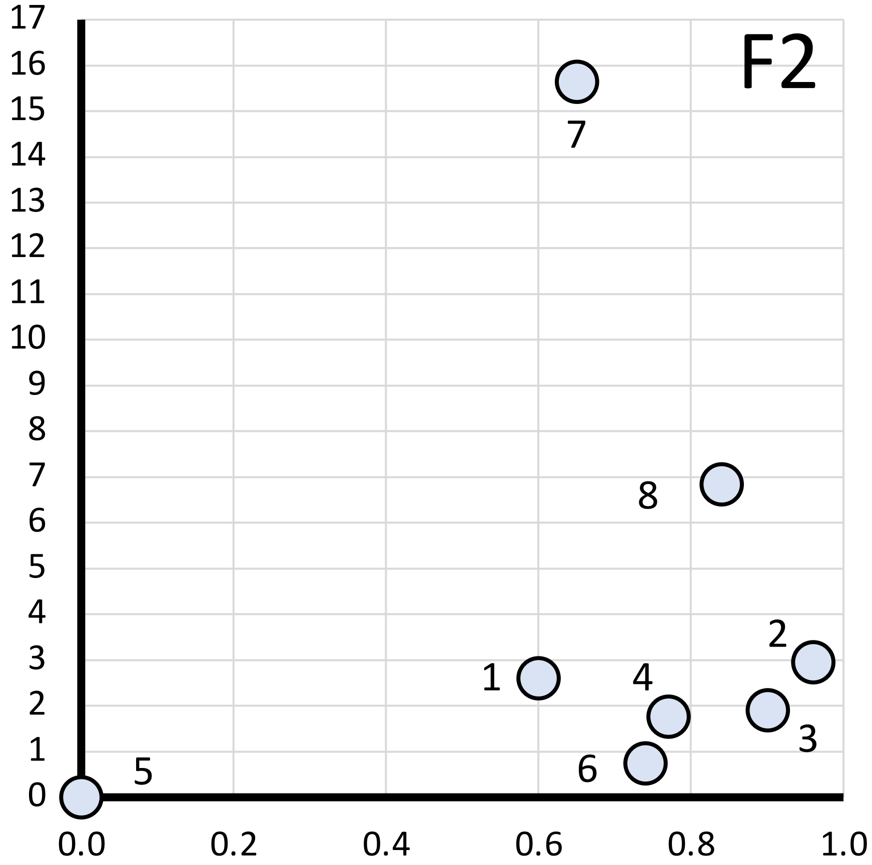

1	2	3	4	5	6	7	8
4.01	2.74	2.28	2.79	0	1.53	18	9.24
0.01	0.74	0.28	0.79	0	0.53	0	0.24

1	2	3	4	5	6	7	8
2.6	2.96	1.9	1.77	0	0.74	15.65	6.84
0.6	0.96	0.9	0.77	0	0.74	0.65	0.84

Breast muscles

Thigh muscles

Layer

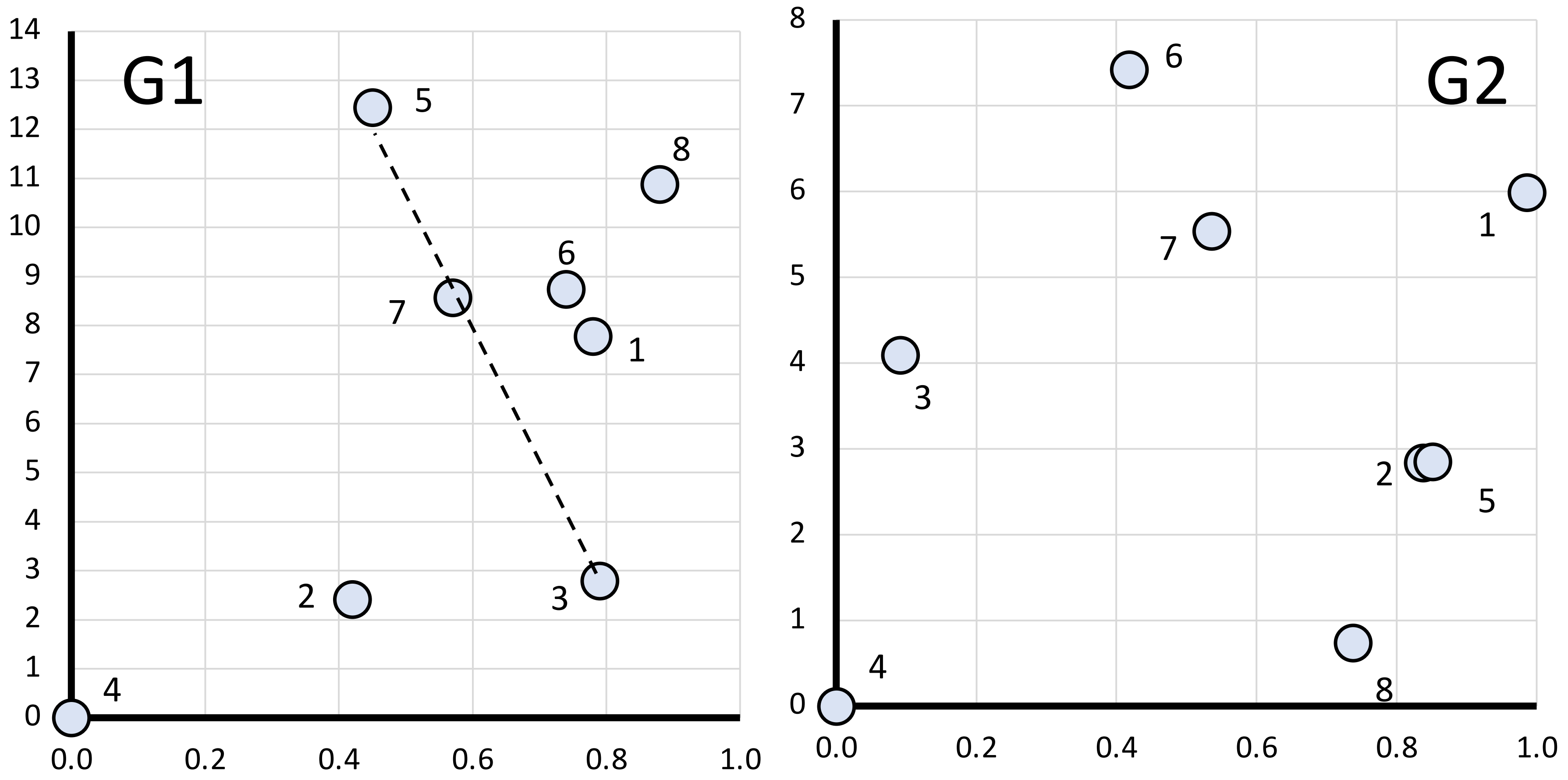

1	2	3	4	5	6	7	8
7.78	2.42	2.79	0	12.45	8.74	8.57	10.88
0.78	0.42	0.79	0	0.45	0.74	0.57	0.88

1	2	3	4	5	6	7	8
5.99	2.84	4.09	0.00	2.85	7.42	5.54	0.74
0.986295	0.838194	0.091117	0	0.851449	0.418272	0.536296	0.73779

Breast muscles

Thigh muscles

Figure S4. Fractal portraits of gene expression in the breast (**A1–G1**) and thigh (**A2–G2**) muscles of embryos in the chicken breeds studied. The *X*-axis shows the fractional parts of the shifted gene expression levels, and the *Y*-axis shows the full values of the shifted gene expression levels (as shown below each plot). The numbers near the dots correspond to the gene numbering in Table S13. The dotted lines show that the corresponding genes belong to gene expression fractals, reflecting the fractal bioconsolidation of these genes.
