# Supplementary material for: Features of Fractal Conformity and Bioconsolidation in the Early Myogenesis Gene Expression and Their Relationship to the Genetic Diversity of Chicken Breeds
Source: Animals (Basel). 2023 Feb 1;13(3):521. doi: 10.3390/ani13030521 (PMC9913260; doi:10.3390/ani13030521)
Supplement: Supplementary file 1 [file animals-13-00521-s001.zip › Supplementary Figures S1-S3 edited.pdf]

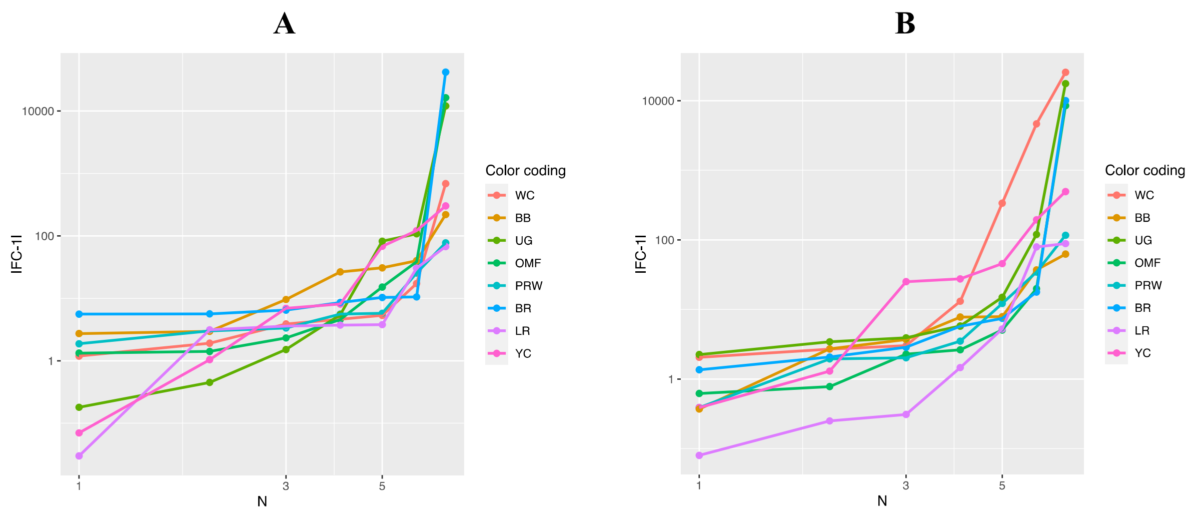


**Figure S1.** Log–log plots of $|FC - 1|(N)$ dependences for gene expression data (Table 2) in the breeds by muscle type. (**A**) Breast muscles; (**B**) thigh muscles.

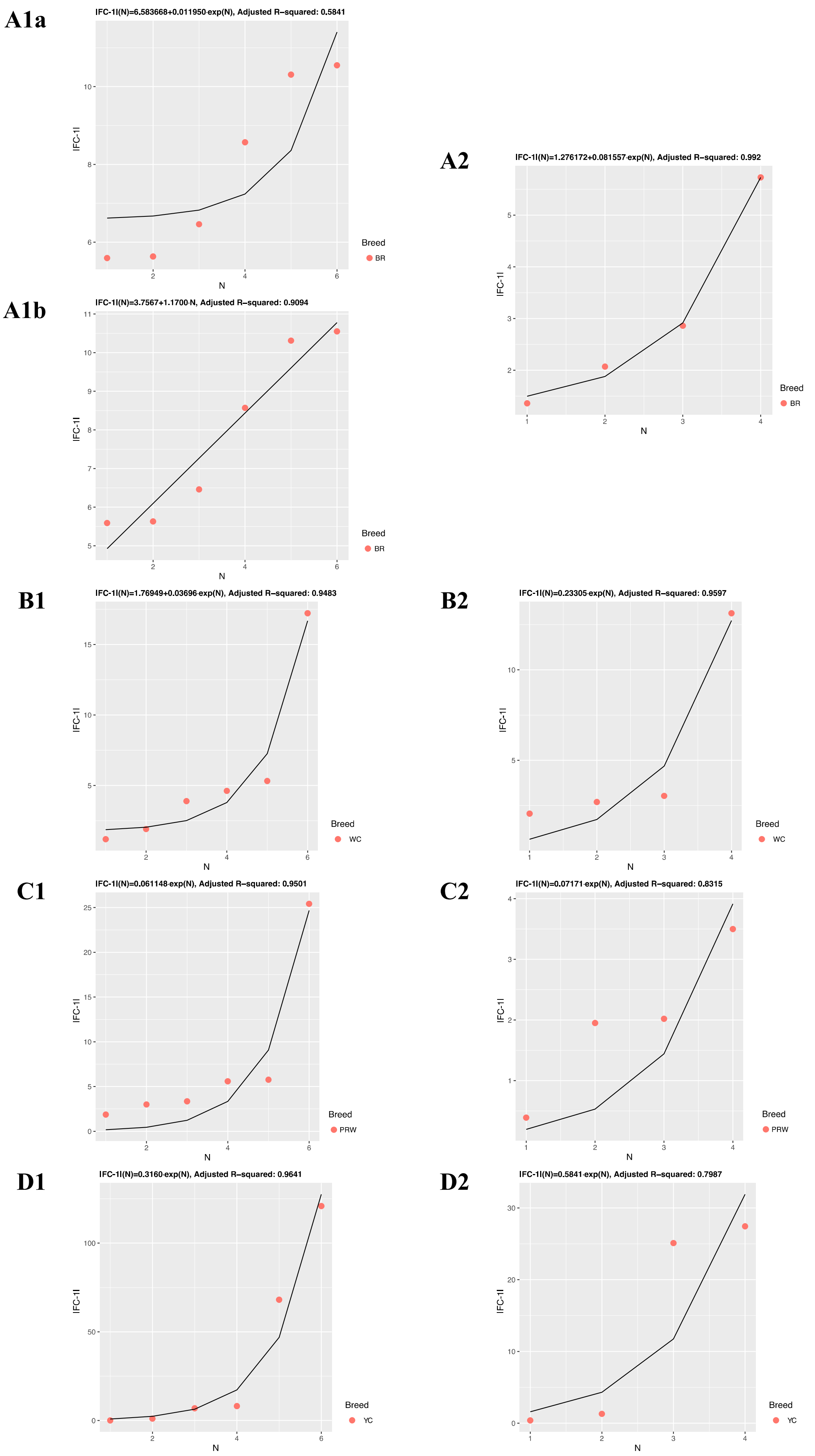
A1a
IFC-1I(N)=6.583668+0.011950·exp(N), Adjusted R−squared: 0.5841
IFC-1I
N
Breed
BR
A1b
IFC-1I(N)=3.7567+1.1700·N, Adjusted R−squared: 0.9094
IFC-1I
N
Breed
BR
A2
IFC-1I(N)=1.276172+0.081557·exp(N), Adjusted R−squared: 0.992
IFC-1I
N
Breed
BR
B1
IFC-1I(N)=1.76949+0.03696·exp(N), Adjusted R−squared: 0.9483
IFC-1I
N
Breed
WC
B2
IFC-1I(N)=0.23305·exp(N), Adjusted R−squared: 0.9597
IFC-1I
N
Breed
WC
C1
IFC-1I(N)=0.061148·exp(N), Adjusted R−squared: 0.9501
IFC-1I
N
Breed
PRW
C2
IFC-1I(N)=0.07171·exp(N), Adjusted R−squared: 0.8315
IFC-1I
N
Breed
PRW
D1
IFC-1I(N)=0.3160·exp(N), Adjusted R−squared: 0.9641
IFC-1I
N
Breed
YC
D2
IFC-1I(N)=0.5841·exp(N), Adjusted R−squared: 0.7987
IFC-1I
N
Breed
YC

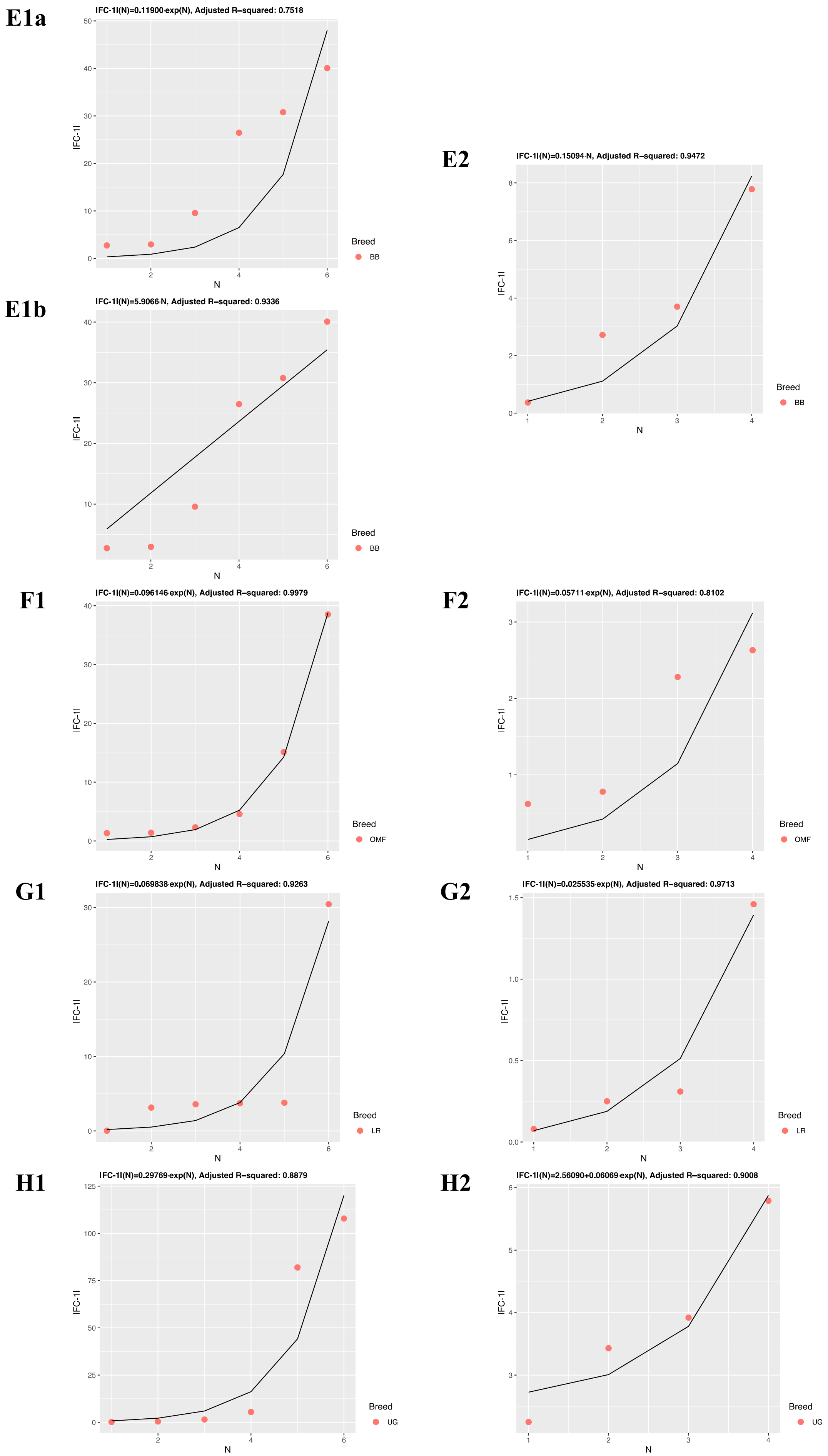


**Figure S2.** Special cases of $|FC-1|(N)$ regressions for different breeds by muscle groups. (**A1–H1**) Breast muscles; (**A2–H2**) thigh muscles. For the breeds BR (**A1b**) and BB (**E1b**), linear models have also been tested.

**A1**

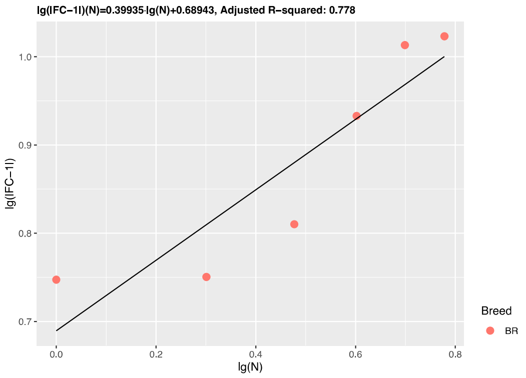


$D$(br) = 0.39935

**B1**

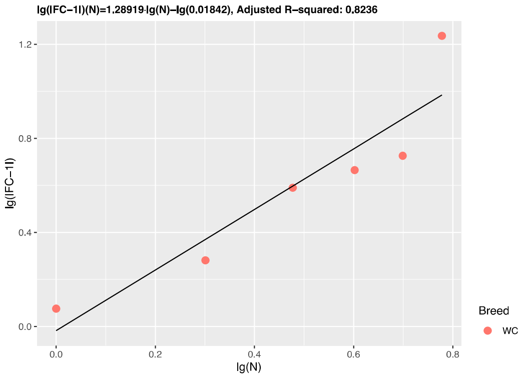


$D$(br) = 1.28919

**C1a**

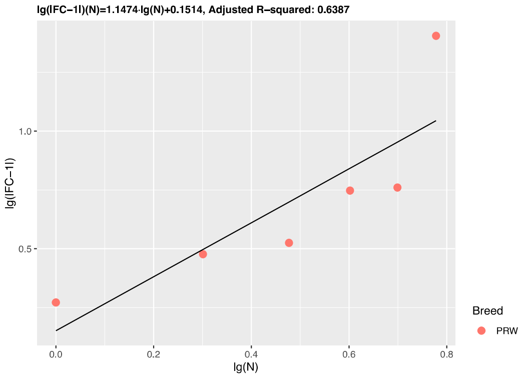


$D$(br) = 1.1474

**C1b**

**A2**

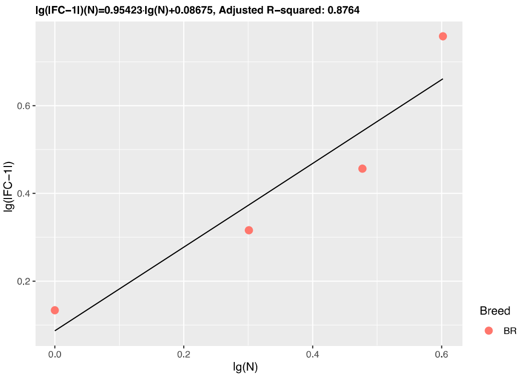


$D$(th) = 0.95423

**B2a**

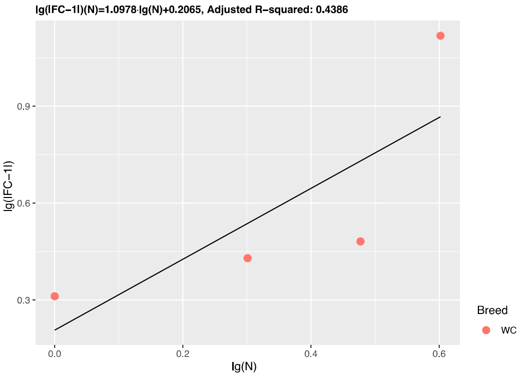


$D$(th) = 1.0978

**B2b**

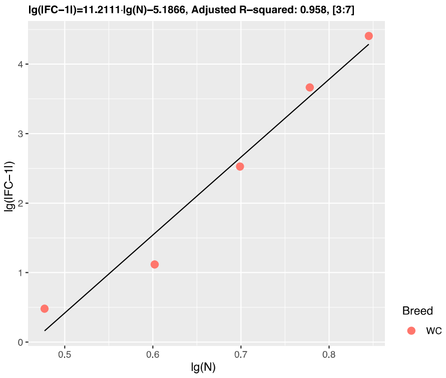


$D$(th) = 11.2111 at $N$[3:7]

**C2**

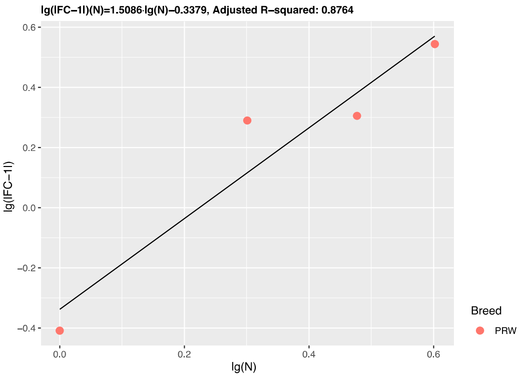


$D$(th) = 1.5086

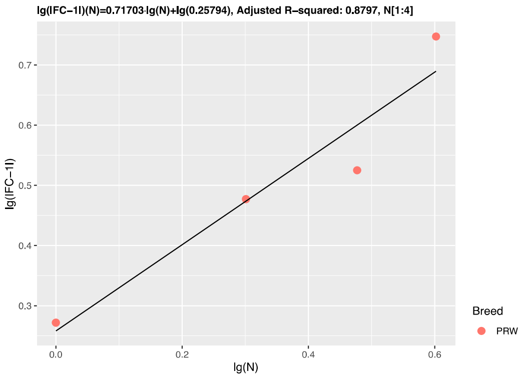


$D$(br) = 0.71703 at $N$[1:4]

**D1**

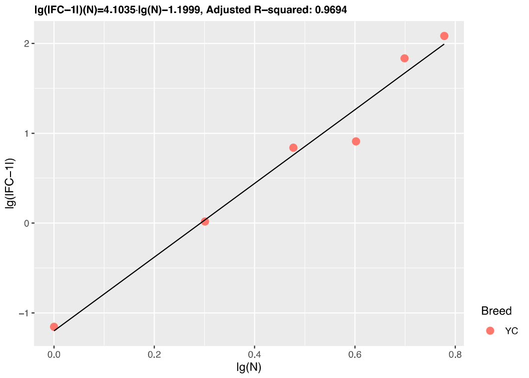


$D$(br) = 4.1035

**D2**

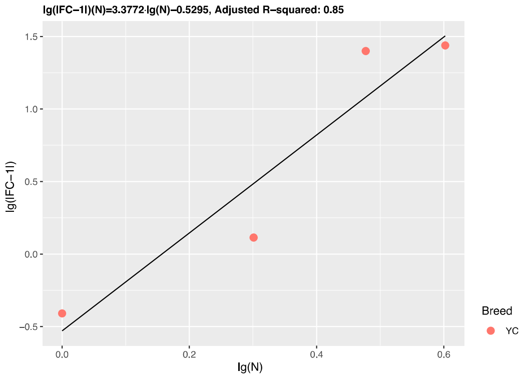


$D$(th) = 3.3772

**E1a**

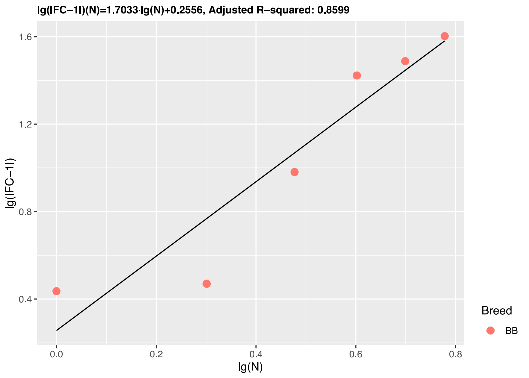


$D$(br) = 1.7033

**E1b**

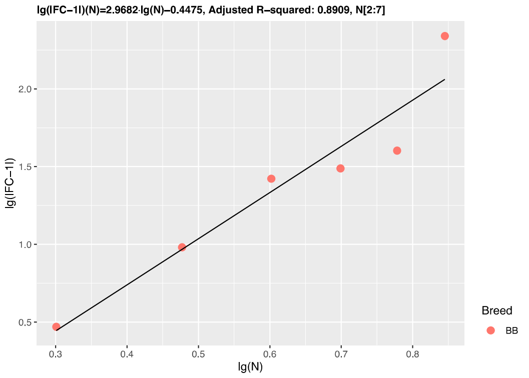


$D$(br) = 2.9682 at $N$[2:7]

**E2**

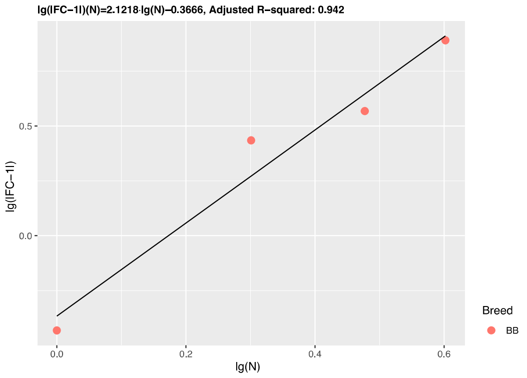


$D$(th) = 2.1218

**F1a**

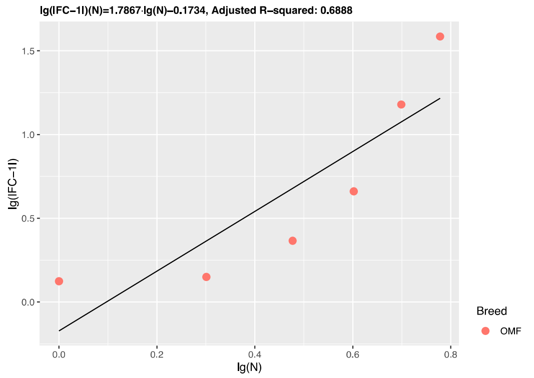


$D$(br) = 1.7867

**F2**

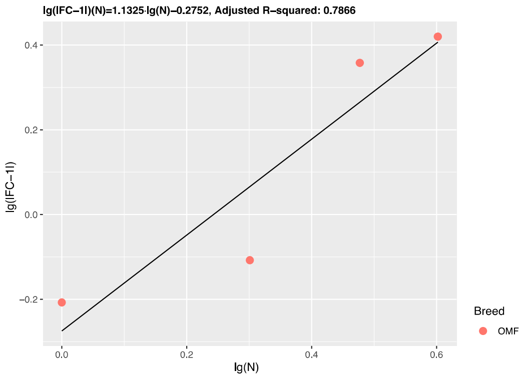


$D$(th) = 1.1325

**F1b**

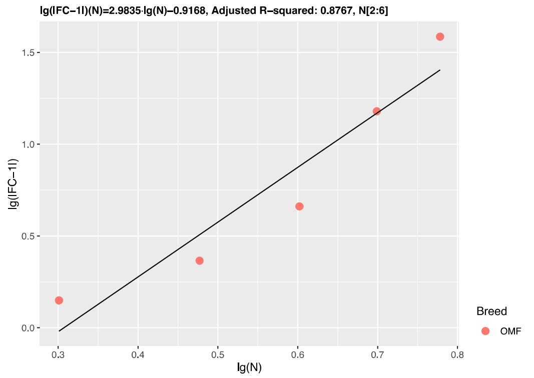


$D$(br) = 2.9835 at $N$[2:6]

**G1**

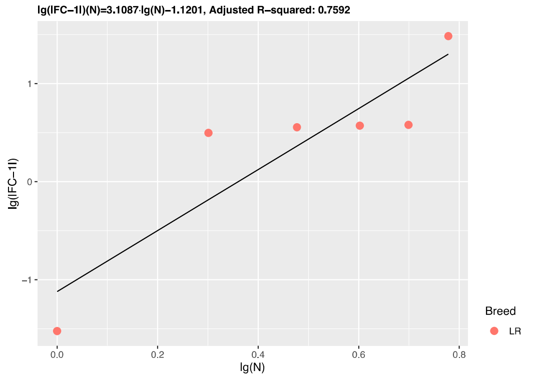


$D$(br) = 3.1087

**G2**

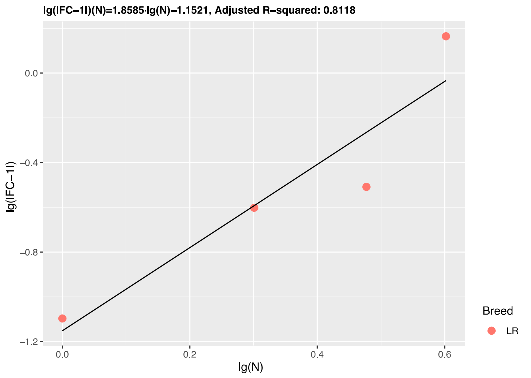


$D$(th) = 1.8585

**H1**

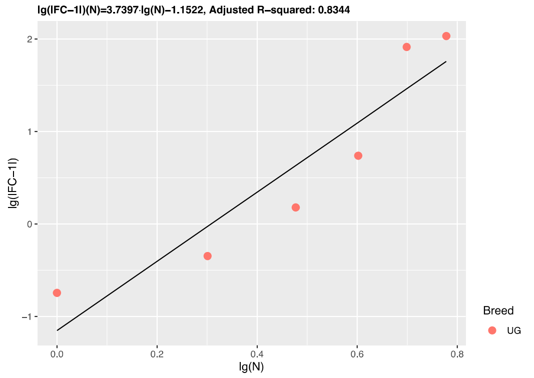


$D$(br) = 3.7397

**H2**

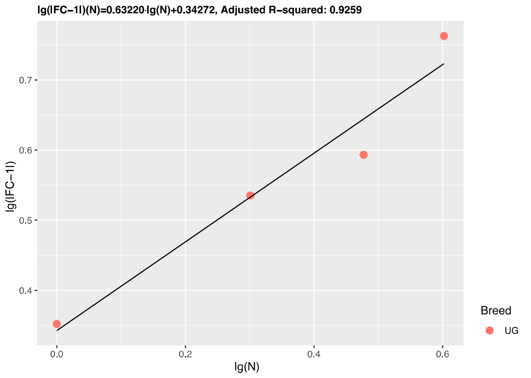


$D$(th) = 0.6322

**Figure S3.** Estimation of fractal dimension coefficients $D$: approximation of dependence lg(|FC − 1|) on lg($N$) and determination of fractality index for gene expression of breast (br) and thigh (th) muscles in different breeds. (**A1–H1**) Breast muscles at $N$[1:6]; (**A2–H2**) thigh muscles at $N$[1:4]. For the breeds WC (**B2b**), PRW (**C1b**), BB (**E1b**) and OMF (**F1b**), different and more optimal ranges of gene ranks ($N$) have also been tested.
