# Supplementary material for: Features of Fractal Conformity and Bioconsolidation in the Early Myogenesis Gene Expression and Their Relationship to the Genetic Diversity of Chicken Breeds
Source: Animals (Basel). 2023 Feb 1;13(3):521. doi: 10.3390/ani13030521 (PMC9913260; doi:10.3390/ani13030521)
Supplement: Supplementary file 1 [file animals-13-00521-s001.zip › Supplementary Information S1 edited.pdf]

Supplementary Information S1: Methodological substantiation for assessing the NO oxidation rate as described in refs [27,47,48,69–74]

Different approaches and methods can be used to estimate the NO oxidation rate. Dinitrosyl iron complexes (DNICs), which form the main pool of NO donors (NOD) in cells, can be determined by electron paramagnetic resonance (EPR). However, most DNICs in tissues are present in non-paramagnetic forms that do not give an EPR signal [69,70]. Methods for the determination of S-nitrosothiols (RSNO) are based on their dissimilation to NO (or NO^+) and its interaction or the formed nitrite with specific reagents. However, these methods do not have high accuracy and specificity [47,71]. It has been shown that the main part of nitric oxide (NO) synthesized in the avian embryo plays a specific role. NO can be accumulated in tissues as part of the NOD compounds or be oxidized to nitrate. This oxidation correlates with the meat production in adult chickens. In broiler embryos, NO is oxidized to nitrate by 90%; in embryos of egg breeds, oxidation is negligible [27]. It has been shown that the degree of NO oxidation is determined by the characteristics of embryonic tissues. Since the degree of NO oxidation varies by no more than 10% within a line or cross [27], it is possible to assume that these tissue features are genetically determined. However, analysis of the heritability of this trait suggests that it is not a specific gene that is inherited. Apparently, the combination of expression of various genes leads to the process(es) associated with the oxidation of nitric oxide to nitrate [27]. The oxidation of NO to nitrate in all avian embryos can be induced by light. Therefore, in all embryos, it is possible to induce the process of NO oxidation [27]. It can be launched either with the help of external factors (light) or with the help of internal, genetically determined factors. It is known that light induces the proliferation and differentiation of myoblasts and satellite cells. It is possible that NO oxidation is associated with these processes [27].

To study the degree of NO oxidation, homogenates of E7 embryos are prepared using a glass homogenizer (8 min, 40 f/min, 6 °C) and an enzyme sensor using a highly sensitive Dithermanal calorimeter (Vaskut-EMG, Hungary). The enzymatic sensor method is based on the reversible inhibition of catalase by all nitroso compounds that initially have an NO^+ group or acquire it under the influence of a number of factors [72]. Halide ions increase the inhibition efficiency by two orders of magnitude. Nitroso compounds lose their inhibitory properties under the action of a number of substances specific to each of their groups [47,48]. In particular, nitro-L-arginine (Sigma-Aldrich, Merck KGaA, Darmstadt, Germany) is used as a NO synthesis blocker. A preparation of arginine (Sigma-Aldrich), a substrate for NO synthesis, is also used. This approach makes it possible to determine the concentration of RSNO, DNICs, nitrite, and nitrosamines with an accuracy of 50 nM [27]. Other catalase inhibitors do not have such features and are not normally found in living tissues in amounts sufficient to introduce significant interference [47,48].

Previous studies [73] found that NO synthesis in an avian embryo starts from the beginning of its development. Already E3 embryo homogenate contains the NOD compounds at a concentration of 140–160 μM, which are represented by RSNO, DNICs, and high-molecular-weight nitro compounds capable of transforming into DNIC (RNO_2). These compounds prolong the physiological lifetime of NO and, according to some studies [69,70,74], directly interact with the physiological target. The concentration of nitrite and nitrosamines in embryonic tissues normally does not exceed 50 nM [47,48]. Therefore, living tissues have a mechanism that minimizes the NO oxidation by oxygen to nitrite and other toxic products. In embryos of broilers and other fast-growing breeds, lines and crosses of chickens, NODs are oxidized to nitrate. Oxidation takes place in the tissues of the embryo.

A study of the composition of nitro- and nitroso compounds in E16 embryos showed that nitrate mainly accumulates in the muscle frame and is removed from there to the allantois [73]. In the embryos of slowly growing breeds, NODs accumulate and are practically not consumed. By the end of the embryonic period, the concentration of nitro and nitroso compounds in the embryo can reach 1 mM. In broiler embryos, up to 90% of these compounds are represented by nitrate, and in embryos of egg breeds, they are NODs [73]. The methodological approaches described here were used to assess the degree of NO oxidation in embryos of various chicken breeds.
