# Supplementary material for: Features of Fractal Conformity and Bioconsolidation in the Early Myogenesis Gene Expression and Their Relationship to the Genetic Diversity of Chicken Breeds
Source: Animals (Basel). 2023 Feb 1;13(3):521. doi: 10.3390/ani13030521 (PMC9913260; doi:10.3390/ani13030521)
Supplement: Supplementary file 1 [file animals-13-00521-s001.zip › Supplementary Information S2 edited.pdf]

**Supplementary Information S2: Search for the relationship between fractal dimension coefficients *D* and body weight of birds**

In the current study, we obtained logarithmic plots (Figures S1A–R) of the gene expression level for the breast and thigh muscles in various chicken breeds depending on the number of genes considered. This is done to show the power-law nature of these dependencies. A distinctive feature of fractal sets, which our data belongs to, is their scale invariance. The distribution of values does not depend on the units in which the values are expressed. Recall that scale invariance allowed us to get rid of negative values by representing the data with the smallest value of zero, calculating the difference between FC and one, and converting the number to the modulus (|FC − 1|). To make sure that the expression level data follow an exponential distribution, we ranked the transformed data and approximated it with an exponent for the breast and thigh muscles and taking into account the breed specificity of gene ranks (Figures 1A,B). For a better approximation, we took data on the expression of 6 and 4 genes for the breast and thigh muscles, respectively. The coefficient $R^2$ for data approximation by the exponent was 0.5485 and 0.4220, respectively.

Let us search for the final models that could explain the relationship of the fractal dimension indices *D* for the breast (Figures S2-1A,B here) and thigh (Figures S2-1C,D) muscles with the 28-day body weight (BW28), and compare them with models for *K* coefficients (Figures S2-1E,F), MGEI (Figures S2-1G) and MGEFDI (Figures S2-1H,I).

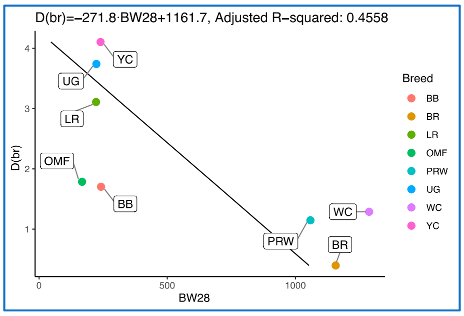


**A**

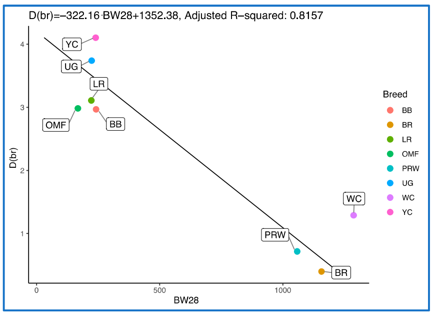


**B**

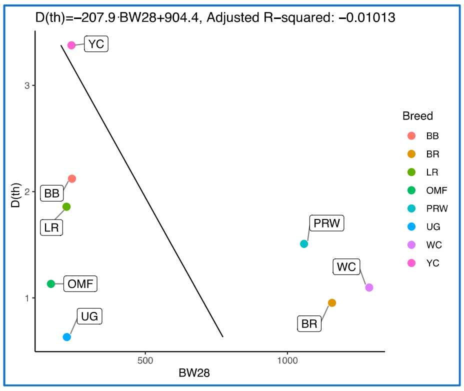


**C**

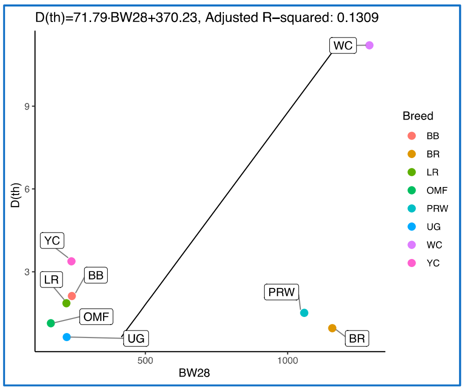


**D**

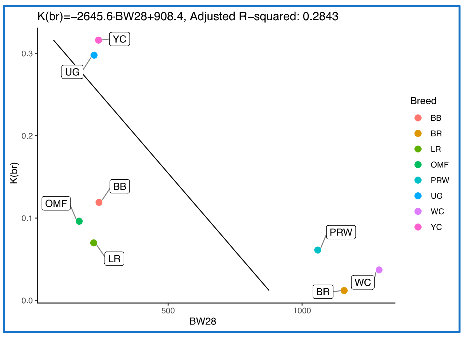


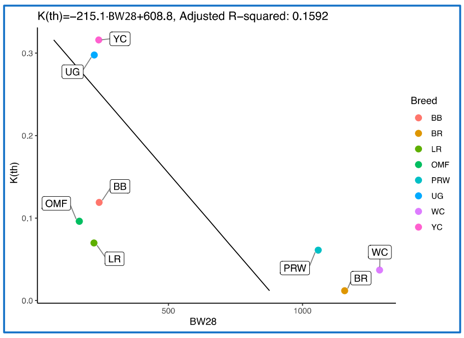

**E** **F**

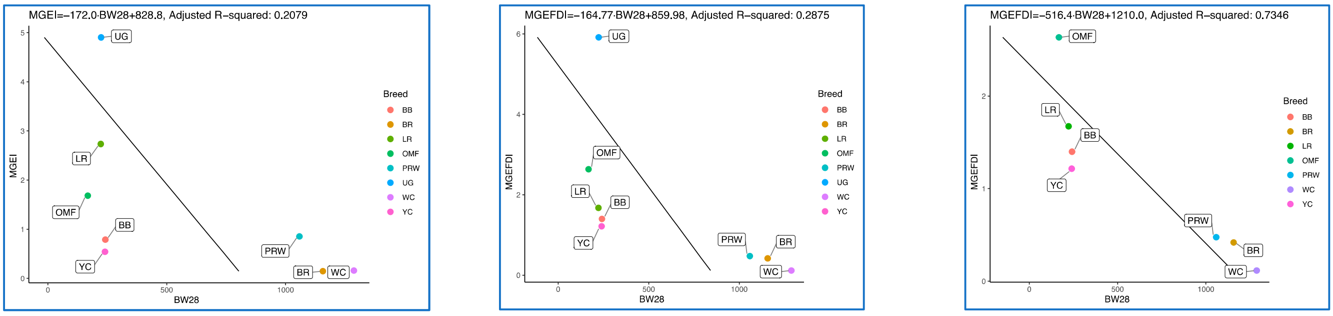


**G** **H** **I**

**Figure SI S2-1.** Linear relationship between body weight of birds at 28 days (BW28) and fractal dimension *D* (**A**–**D**), *K* (**E**, **F**) and MGEI coefficients for gene expression (**G**, **H**, **I**) in the breast (br) and thigh (th) muscles of E14 embryos. (**A**) Model *D*(br) = *f* (BW28), $R^2$ = 0.4558; (**B**) model *D*(br) = *f* (BW28) after adjusting *N* for the BB, OMF and PRW breeds, $R^2$ = 0.8157; (**C**) model D(th) = *f* (BW28), $R^2$ = −0.01013; (**D**) *D*(th) = *f* (BW28) after N correction for WC, $R^2$ = 0.131; (**E**) model *K*(br) = *f* (BW28), $R^2$ = 0.2843; (**F**) model *K*(th) = *f* (BW28), $R^2$ = 0.1592; (**G**) model MGEI = *f* (BW28), $R^2$ = 0.2079; (**H**) model MGEFDI = *f* (BW28) for 8 breeds (of 4 types), $R^2$ = 0.2875; (**I**) model MGEFDI = *f* (BW28) for 7 breeds (of 3 types; with UG excluded), $R^2$ = 0.7346.

Based on the $R^2$ values in the compared composite models, the model with the generally accepted fractal dimension coefficient *D*(br) and manual selection of a straight-line segment for approximation ($R^2$ = 0.8157), as well as the MGEFDI model for 7 breeds (3 types, UG being excluded as an outlier), while the rest of the models had the worst prediction. The general trends in the dependences of indicators and BW28 remain, however, in all models. The main disadvantage of the *D* search method is the need for a manual approach to each data set to determine the most direct sections. In addition, it is not possible to find adequate *D* values for all muscle groups due to limited data.
