# Supplementary material for: Features of Fractal Conformity and Bioconsolidation in the Early Myogenesis Gene Expression and Their Relationship to the Genetic Diversity of Chicken Breeds
Source: Animals (Basel). 2023 Feb 1;13(3):521. doi: 10.3390/ani13030521 (PMC9913260; doi:10.3390/ani13030521)
Supplement: Supplementary file 1 [file animals-13-00521-s001.zip › Supplementary Information S3.pdf]

Supplementary Information S3: Extended discussion on the developed mathematical models and indices

An unequivocal answer to the question of which individual myogenesis genes make the main contribution to the formation of the *K* and MGEI coefficients has not been received here (see the example for *GHR* in Table S6). According to the rank distributions of gene expression (Tables S1 and S2) from which these coefficients were derived, expression of certain key genes that is characteristic of breed types cannot be identified as this requires validation by a formal test. The coefficients *K*(br) (for the breast muscles) and *K*(th) (for the thigh muscles), derived as the slopes of the function (1), apparently reflect the coordinated network of the genes. The smaller the difference in the expression of extreme genes in their rank distribution, the smaller the slope of the exponential curve describing them, and the better the network of the studied genes is coordinated, which is reflected in an increased body weight gain and may be a characteristic feature for a certain type of breed. Breed types with lower body weight gain (egg, game and dual purpose breeds) have higher *K*(br) and MGEI coefficients (Table 3), showing a significant difference between gene expressions. Along with weakly active genes, there are genes that are highly expressed during this development stage. Thus, the network of a set of genes in the embryonic period can predetermine the physiological development of muscles in the postembryonic period.

The MGEI coefficient value grows from meat breeds (through dual purpose ones) to egg and game breeds, since it reflects the specifics of the conformity of the myogenesis gene network in different muscle types and chicken breed types. The stronger the relationship between gene expressions, the smaller the difference between them in the expression of extreme genes in their rank distribution. The position of the UG game breed looks very unusual as a completely isolated breed, moreover, with the highest possible MGEI coefficient, which is 1.8 times higher than that of the egg breed. This observation seems to require further consideration. From the phenotypic data obtained, it is also clear that the egg, dual purpose and game breeds are not characterized by the same body muscularity and GR compared to meat breeds. This means that commercial meat (broiler) breeds have the highest rates of body weight gain due to long-term artificial selection for meat traits. The game breed was not subject to such selection. For any game breed, the most important are fighting qualities with a fairly light body weight that ensures the mobility of a rooster in arranged (in the past) fights. Therefore, the increase in body weight of game chickens is more consistent with that of egg chickens. Then, the data of Table 3 with MGEI values can be quite well interpreted in the case of the game breed with the breed features described above.

It can be assumed that the success in the development of embryos and chicks and their more intensive weight gain are determined not only by the intensities of gene expression, but to a greater extent by the proximity of gene expression levels to log arithmetic fractal series. Obviously, if some protein synthesized by the gene is missing in the body, the overall development of the bird may slow down and its weight gain in the breast or thigh muscles will slow down. Therefore, a certain dynamic ratio of gene expression seems to be maintained throughout the development of a bird.
