# Supplementary material for: Features of Fractal Conformity and Bioconsolidation in the Early Myogenesis Gene Expression and Their Relationship to the Genetic Diversity of Chicken Breeds
Source: Animals (Basel). 2023 Feb 1;13(3):521. doi: 10.3390/ani13030521 (PMC9913260; doi:10.3390/ani13030521)
Supplement: Supplementary file 1 [file animals-13-00521-s001.zip › Supplementary Tables S1-S14 edited.pdf]

Table S1. Rank distribution of the considered breast muscle genes according to the increasing level of their differential expression (after |FC − 1| transformation).

Breed	Serial gene rank number (*N*)						
	**1**	**2**	**3**	**4**	**5**	**6**	**7**
Broiler	5.59 (*MEF2C*)	**5.63 (*GHR*)**	6.46 (*MYOG*)	8.57 (*MYF5*)	10.31 (*MYOD1*)	10.55 (*MSTN*)	41761.0 (*MYH1*)
White Cornish	1.19 (*MYOD1*)	1.91 (*MEF2C*)	3.89 (*MSTN*)	4.62 (*GHR*)	5.32 (*MYOG*)	17.22 (*MYH1*)	686.00 (*MYF5*)
Plymouth Rock White	1.87 (*MYOD1*)	3.00 (*MEF2C*)	3.35 (*GHR*)	5.59 (*MSTN*)	5.76 (*MYF5*)	25.42 (*MYH1*)	77.25 (*MYOG*)
Yurlov Crower	0.07 (*MYH1*)	1.04 (*MYOG*)	6.90 (*MYF5*)	8.11 (*MYOD1*)	68.10 (*GHR*)	120.90 (*MSTN*)	301.30 (*MEF2C*)
Brahma Buff	2.73 (*MYH1*)	2.95 (*MYOG*)	9.57 (*MYF5*)	26.46 (*MYOD1*)	30.78 (*GHR*)	40.07 (*MSTN*)	218.80 (*MEF2C*)
Orloff Mille Fleur	1.33 (*MEF2C*)	1.41 (*MSTN*)	2.32 (*GHR*)	4.58 (*MYOG*)	15.11 (*MYOD1*)	38.53 (*MYF5*)	16271.0 (*MYH1*)
Layer	0.03 (*MYOG*)	3.14 (*MEF2C*)	3.59 (*MYOD1*)	3.72 (*MSTN*)	3.79 (*GHR*)	30.45 (*MYH1*)	67.26 (*MYF5*)
Uzbek Game	0.18 (*MSTN*)	0.45 (*MEF2C*)	1.51 (*GHR*)	5.47 (*MYF5*)	82.01 (*MYOD1*)	107.90 (*MYOG*)	11991.0 (*MYH1*)

In all breeds, the specific color indicates the same genes taken to build a regression curve for each breed. However, only one gene (*GHR*) was included in the regression curves for all breeds. Rank 7 genes were not taken to plot the curves in Figures 1A, S1A and 2A.

Table S2. Rank distribution of the considered thigh muscle genes according to the increasing level of their differential expression (after |FC − 1| transformation).

Breed	Serial gene rank number (*N*)						
	**1**	**2**	**3**	**4**	**5**	**6**	**7**
Broiler	1.36 (*MEF2C*)	2.07 (*GHR*)	2.86 (*MSTN*)	5.73 (*MYOG*)	7.45 (*MYF5*)	17.77 (*MYOD1*)	10021.00 (*MYH1*)
White Cornish	2.05 (*GHR*)	2.69 (*MEF2C*)	3.03 (*MSTN*)	13.13 (*MYOD1*)	336.46 (*MYH1*)	4641.00 (*MYOG*)	25531.00 (*MYF5*)
Plymouth Rock White	0.39 (*MYOG*)	1.95 (*GHR*)	2.02 (*MEF2C*)	3.50 (*MSTN*)	12.18 (*MYOD1*)	34.36 (*MYF5*)	116.36 (*MYH1*)
Yurlov Crower	0.39 (*MYOG*)	1.30 (*MYH1*)	25.10 (*GHR*)	27.44 (*MYOD1*)	45.53 (*MSTN*)	194.36 (*MYF5*)	493.56 (*MEF2C*)
Brahma Buff	0.37 (*MYH1*)	2.72 (*GHR*)	3.70 (*MYOG*)	7.78 (*MYOD1*)	7.86 (*MSTN*)	37.02 (*MYF5*)	62.39 (*MEF2C*)
Orloff Mille Fleur	0.62 (*GHR*)	0.78 (*MEF2C*)	2.28 (*MSTN*)	2.63 (*MYOG*)	5.06 (*MYOD1*)	19.90 (*MYF5*)	8482.00 (*MYH1*)
Layer	0.08 (*MYOD1*)	0.25 (*MSTN*)	0.31 (*GHR*)	1.46 (*MEF2C*)	5.23 (*MYH1*)	79.25 (*MYOG*)	88.43 (*MYF5*)
Uzbek Game	2.25 (*MSTN*)	3.43 (*MYF5*)	3.92 (*GHR*)	5.79 (*MEF2C*)	14.93 (*MYOD1*)	119.60 (*MYOG*)	17561.00 (*MYH1*)

In all breeds, the specific color indicates the same genes taken to build a regression curve for each breed. However, only one gene (*GHR*) was included in the regression curves for all breeds. Genes of the 5th, 6th and 7th ranks were not taken to plot the curves in Figures 1B, S1B and 2B.

Table S3. Ordered (by descending) breed-specific values of the *K*(br) coefficient in thigh muscles compared to body weight of chicks at 14 and 28 days.

Trait	Yurlov Crower	Uzbek Game	Brahma Buff	Orloff Mille Fleur	Layer	Plymouth Rock White	White Cornish	Broiler
K(br)	0.316	0.298	0.119	0.096	0.070	0.061	0.037	0.012
Body weight at 14 days	101.3	92.4	107.5	93.1	79.8	265.2	291.7	305.0
Body weight at 28 days	240.0	223.7	241.7	167.8	222.4	1058.4	1287.5	1157.0

Table S4. Relationships between *K* coefficients and phenotypic/metabolic traits when comparing Pearson and Spearman correlations (without and with natural log normalization).

Trait[1]	*K*(br)				*K*(th)				*K*(br)/*K*(th)			
	Pearson / Spearman correlation (no normalization)		Pearson / Spearman correlation (after normalization)		Pearson / Spearman correlation (no normalization)		Pearson / Spearman correlation (after normalization)		Pearson / Spearman correlation (no normalization)		Pearson / Spearman correlation (after normalization)	
	Correlation	*p*-value	Correlation	*p*-value	Correlation	*p*-value	Correlation	*p*-value	Correlation	*p*-value	Correlation	*p*-value
BW1	-0.5061792<br>-0.7619048	0.2006<br>0.03676	-0.6729326<br>-0.7619048	0.06742<br>0.03676	-0.1148909<br>0.1904762	0.7865<br>0.1904762	0.01056685<br>0.1904762	0.9802<br>0.6646	-0.305616<br>-0.452381	0.4617<br>0.2675	0.01056685<br>0.1904762	0.9802<br>0.6646
BW14	-0.6225808<br>-0.6428571	0.09923<br>0.09618	-0.7555818<br>-0.6428571	0.03014<br>0.09618	-0.08462337<br>0.6190476	0.8421<br>0.115	0.1951829<br>0.6190476	0.6432<br>0.115	-0.599050<br>-0.88095246	0.1166<br>0.007242	-0.7991158<br>-0.8809524	0.01734<br>0.007242
BW2	-0.621709<br>-0.5952381	0.09984<br>0.1323	-0.7463845<br>-0.5952381	0.03342<br>0.1323	-0.0800549<br>-0.0800549	0.8505<br>0.6666667	0.1539887<br>0.6666667	0.7158<br>0.08309	-0.5666495<br>-0.7857143	0.1431<br>0.02793	-0.7593802<br>-0.7857143	0.02885<br>0.02793
GR2wk	-0.6219097<br>-0.7142857	0.0997<br>0.05759	-0.7367275<br>-0.7142857	0.03709<br>0.05759	-0.08242679<br>0.4761905	0.8461<br>0.2431	0.2273622<br>0.4761905	0.5882<br>0.2431	-0.6306709<br>-0.8095238	0.09364<br>0.02178	-0.7367275<br>-0.7142857	0.03709<br>0.05759
GR4wk	-0.6210358<br>-0.5952381	0.1003<br>0.1323	-0.7367275<br>-0.7142857	0.03709<br>0.05759	-0.08326223<br>0.6666667	0.8446<br>0.08309	0.2273622<br>0.4761905	0.5882<br>0.2431	-0.5775628<br>-0.7857143	0.1338<br>0.02793	-0.7367275<br>-0.7142857	0.03709<br>0.05759
NOD	0.1345843<br>0.4047619	0.7507<br>0.3268	0.4582007<br>0.4047619	0.2535<br>0.3268	0.2174358<br>0.04761905	0.605<br>0.9349	-0.04269044<br>0.04761905	0.9201<br>0.9349	-0.04850956<br>0.1904762	0.9092<br>0.6646	0.4582007<br>0.4047619	0.2535<br>0.3268
Nitrate	-0.1039682<br>-0.4312443	0.8065<br>0.2861	-0.2660855<br>-0.4312443	0.5241<br>0.2861	-0.1509346<br>0.3678261	0.7213<br>0.37	0.1435787<br>0.3678261	0.7345<br>0.37	0.03430042<br>-0.4692953	0.9357<br>0.2407	-0.2660855<br>-0.4312443	0.5241<br>0.2861
NO oxidation	-0.0945139<br>-0.5628843	0.8238<br>0.1463	-0.2904644<br>-0.5628843	0.4852<br>0.1463	-0.1623345<br>0.0958101	0.7009<br>0.8215	0.1158026<br>0.0958101	0.7848<br>0.8215	0.04185864<br>-0.3113828	0.9216<br>0.4528	-0.2904644<br>-0.5628843	0.4852<br>0.1463
NOD category	No correlation (ANOVA, $p > 0.05$)											
Nitrate category	No correlation (ANOVA, $p > 0.05$)											
NO oxidation category	No correlation (ANOVA, $p > 0.05$)											

Breed type category	$p = 0.09$	after normalization by natural logarithm, $p = 0.0244$ (ANOVA)	$p = 0.756$	$p = 0.155$

[1] Abbreviations: BW1, BW14 and BW28, body weight at 1, 14 and 28 days; NOD, content of NO donors; Nitrate, nitrate content; NO oxidation, rate of NO oxidation to nitrate; GR2wk and GR4wk, growth rate for 2 and 4 weeks. The green color represents the results of calculations for the Spearman's correlation. Significant ($p < 0.05$) and strongest associations are highlighted in yellow.

Table S5. Indicators *K*(br), *K*(th) and *K*(br)/*K*(th) depending on the utility type of breeds.

Breed	Type	*K*(br)	*K*(th)	*K*(br)/*K*(th)
No sorting				
Broiler	Meat	0.011950	0.081557	0.146523290459433
White Cornish	Meat	0.03696	0.23305	0.158592576700279
Plymouth Rock White	Dual purpose (meat but also used for egg production)	0.061148	0.07171	0.85271231348487
Yurlov Crower	Dual purpose (but has a game ancestor)	0.3160	0.5841	0.54100325286766
Brahma Buff	Dual purpose (but has a game ancestor)	0.11900	0.15094	0.788392738836624
Orloff Mille Fleur	Dual purpose (but has a game ancestor)	0.096146	0.05711	1.6835230257398
Layer	Egg	0.069838	0.025535	2.73499118856472
Uzbek Game	Game	0.29769	0.06069	4.90509144834404
Sorted in ascending order by K(br)				
Broiler	Meat	0.011950	0.081557	0.146523290459433
White Cornish	Meat	0.03696	0.23305	0.158592576700279
Plymouth Rock White	Dual purpose (meat but also used for egg production)	0.061148	0.07171	0.85271231348487
Layer	Egg	0.069838	0.025535	2.73499118856472
Orloff Mille Fleur	Dual purpose (but has a game ancestor)	0.096146	0.05711	1.6835230257398
Brahma Buff	Dual purpose (but has a game ancestor)	0.11900	0.15094	0.788392738836624
Uzbek Game	Game	0.29769	0.06069	4.90509144834404
Yurlov Crower	Dual purpose (but has a game ancestor)	0.3160	0.5841	0.54100325286766
Sorted in ascending order by K(th)				
Layer	Egg	0.069838	0.025535	2.73499118856472
Orloff Mille Fleur	Dual purpose (but has a game ancestor)	0.096146	0.05711	1.6835230257398
Uzbek Game	Game	0.29769	0.06069	4.90509144834404
Plymouth Rock White	Dual purpose (meat but also used for egg production)	0.061148	0.07171	0.85271231348487
Broiler	Meat	0.011950	0.081557	0.146523290459433
Brahma Buff	Dual purpose (but has a game ancestor)	0.11900	0.15094	0.788392738836624
White Cornish	Meat	0.03696	0.23305	0.158592576700279
Yurlov Crower	Dual purpose (but has a game ancestor)	0.3160	0.5841	0.54100325286766
Sorted in ascending order by K(br)/K(th)				
Broiler	Meat	0.011950	0.081557	0.146523290459433
White Cornish	Meat	0.03696	0.23305	0.158592576700279
Yurlov Crower	Dual purpose (but has a game ancestor)	0.3160	0.5841	0.54100325286766
Brahma Buff	Dual purpose (but has a game ancestor)	0.11900	0.15094	0.788392738836624
Plymouth Rock White	Dual purpose (meat but also used for egg production)	0.061148	0.07171	0.85271231348487
Orloff Mille Fleur	Dual purpose (but has a game ancestor)	0.096146	0.05711	1.6835230257398
Layer	Egg	0.069838	0.025535	2.73499118856472
Uzbek Game	Game	0.29769	0.06069	4.90509144834404

For groups highlighted in yellow and green, color coding allows you to see if the order of breed types is preserved when ranking by different *K*.

Table S6. Coefficients and FC values of the *GHR* gene when sorted by MGEI* and depending on the utility type of breeds.

**Breed**	**Type**	***K*(br)**	***K*(th)**	**MGEI**	**FC (*GHR*)**
Broiler	Meat	0.011950	0.081557	0.146523290459433	6.63
White Cornish	Meat	0.03696	0.23305	0.158592576700279	5.62
Yurlov Crower	Dual purpose	0.3160	0.5841	0.54100325286766	69.1
Brahma Buff	Dual purpose	0.11900	0.15094	0.788392738836624	31.78
Plymouth Rock White	Dual purpose	0.061148	0.07171	0.85271231348487	4.35
Orloff Mille Fleur	Dual purpose	0.096146	0.05711	1.6835230257398	3.32
Layer	Egg	0.069838	0.025535	2.73499118856472	4.79
Uzbek Game	Game	0.29769	0.06069	4.90509144834404	2.51

* MGEI = *K*(br)/*K*(th).

Table S7. Dynamics of changes in the body weight of chicks over a 4-week period.

Breed	Body weight, g		
	**1 day**	**14 days**	**28 days**
Broiler	47.5	305.0	1157.0
White Cornish	49.3	291.7	1287.5
Plymouth Rock White	44.9	265.2	1058.4
Yurlov Crower	39.0	101.3	240
Brahma Buff	38.2	107.5	241.7
Orloff Mille Fleur	35.5	93.1	167.8
Layer	42.4	79.8	222.4
Uzbek Game	41.3	92.4	223.7

The color combines breeds with approximately the same growth rate.

Table S8. Dynamics of changes in body weight and growth rate of chicks over 2- and 4-week periods in comparison with the FC values of the *GHR* gene.

Breed	Body weight, g			Growth rate		FC (*GHR*)
	1 day	14 days	28 days	2 weeks	4 weeks	
Layer	42.4	79.8	222.4	1.88207547	5.24528302	4.79
Uzbek Game	41.3	92.4	223.7	2.23728814	5.41646489	2.51
Yurlov Crower	39.0	101.3	240.0	2.5974359	6.15384615	69.1
Orloff Mille Fleur	35.5	93.1	167.8	2.62253521	4.72676056	3.32
Brahma Buff	38.2	107.5	241.7	2.81413613	6.32722513	31.78
Plymouth Rock White	44.9	265.2	1058.4	5.9064588	23.5723831	4.35
White Cornish	49.3	291.7	1287.5	5.9168357	26.1156187	5.62
Broiler	47.5	305.0	1157.0	6.42105263	24.3578947	6.63

Note: Data sorted in ascending 2-week growth rate.

Table S9. Spearman correlation coefficient values for 11 indicators of growth, NO metabolism and myogenesis across all the breeds studied.

Trait[1]	BW1	BW14	BW28	GR2wk	GR4wk	NOD	Nitrate	NO oxidation	*K*(br)	*K*(th)	MGEI*
BW1	1.0000000	0.5714286	0.7619048	0.5238095	0.7619048	-0.33333333	0.5707646	0.6227657	-0.7619048	0.19047619	-0.4523810
BW14	0.5714286	1.0000000	0.9047619	0.9761905	0.9047619	-0.33333333	0.5580809	0.5389318	-0.6428571	0.61904762	-0.8809524
BW28	0.7619048	0.9047619	1.0000000	0.8333333	1.0000000	-0.33333333	0.6722338	0.6347419	-0.5952381	0.66666667	-0.7857143
GR2wk	0.5238095	0.9761905	0.8333333	1.0000000	0.8333333	-0.40476190	0.5580809	0.5628843	-0.7142857	0.47619048	-0.8095238
GR4wk	0.7619048	0.9047619	1.0000000	0.8333333	1.0000000	-0.33333333	0.6722338	0.6347419	-0.5952381	0.66666667	-0.7857143
NOD	-0.3333333	-0.3333333	-0.3333333	-0.4047619	-0.3333333	1.00000000	-0.8624887	-0.8982197	0.4047619	0.04761905	0.1904762
Nitrate	0.5707646	0.5580809	0.6722338	0.5580809	0.6722338	-0.86248869	1.0000000	0.8931885	-0.4312443	0.36782606	-0.4692953
NO oxidation	0.6227657	0.5389318	0.6347419	0.5628843	0.6347419	-0.89821970	0.8931885	1.0000000	-0.5628843	0.09581010	-0.3113828
K(br)	-0.7619048	-0.6428571	-0.5952381	-0.7142857	-0.5952381	0.40476190	-0.4312443	-0.5628843	1.0000000	0.09523810	0.4761905
K(th)	0.1904762	0.6190476	0.6666667	0.4761905	0.6666667	0.4761905	0.3678261	0.0958101	0.0952381	1.0000000	-0.7619048

MGEI	-0.4523810	-0.8809524	-0.7857143	-0.8095238	-0.7857143	0.19047619	-0.4692953	-0.3113828	0.4761905	-0.76190476	1.0000000

[1] BW1, BW14 и BW28, body weight at 1, 14 and 28 days; GR2wk and GR4wk, growth rate for 2 and 4 weeks; NOD, content of NO donors; Nitrate, content of nitrate; NO oxidation, rate of NO oxidation to nitrate; K(br), coefficient K in the breast muscles; K(th), K coefficient in the thigh muscles; MGEI, myogenesis gene expression index, equal to K(br)/K(th) ratio. Yellow highlighted are significant correlation values ($p < 0.05$; see Table 14).

Table S10. Spearman correlation *p*-values for 11 indicators shown in Supplementary Table S9.

Trait[1]	BW1	BW14	BW28	GR2wk	GR4wk	NOD	Nitrate	NO oxidation	*K*(br)	*K*(th)	MGEI*
BW1	0.00000000	0.1511408730	0.03675595	0.1966269841	0.03675595	0.427876984	0.139525273	0.099100808	0.03675595	0.66458333	0.267460317
BW14	0.15114087	0.0000000000	0.004563492	0.0003968254	0.004563492	0.427876984	0.150568177	0.168118035	0.09618056	0.11498016	0.007242063
BW28	0.03675595	0.0045634921	0.000000000	0.0153769841	0.00004960317	0.427876984	0.067808887	0.090890436	0.13229167	0.08308532	0.027926587
GR2wk	0.19662698	0.0003968254	0.01537698	0.0000000000	0.01537698	0.326835317	0.150568177	0.146331533	0.05758929	0.24305556	0.021775794
GR4wk	0.03675595	0.0045634921	0.00004960317	0.0153769841	0.000000000	0.427876984	0.067808887	0.090890436	0.13229167	0.08308532	0.027926587
NOD	0.42787698	0.4278769841	0.4278770	0.3268353175	0.4278770	0.000000000	0.005848633	0.002438797	0.32683532	0.93487103	0.664583333
Nitrate	0.13952527	0.1505681771	0.06780889	0.1505681771	0.06780889	0.005848633	0.000000000	0.002807615	0.28607285	0.37000799	0.240731006
NO oxidation	0.09910081	0.1681180353	0.09089044	0.1463315326	0.09089044	0.002438797	0.002807615	0.000000000	0.14633153	0.82145240	0.452798757
K(br)	0.03675595	0.0961805556	0.1322917	0.0575892857	0.1322917	0.326835317	0.286072854	0.146331533	0.000000000	0.84012897	0.243055556
K(th)	0.66458333	0.1149801587	0.08308532	0.2430555556	0.08308532	0.93487103	0.37000799	0.82145240	0.84012897	0.000000000	0.03675595
MGEI	0.26746032	0.0072420635	0.02792659	0.0217757937	0.02792659	0.664583333	0.240731006	0.452798757	0.24305556	0.03675595	0.000000000

[1] BW1, BW14 и BW28, body weight at 1, 14 and 28 days; GR2wk and GR4wk, growth rate for 2 and 4 weeks; NOD, content of NO donors; Nitrate, content of nitrate; NO oxidation, rate of NO oxidation to nitrate; K(br), coefficient K in the breast muscles; K(th), K coefficient in the thigh muscles; MGEI, myogenesis gene expression index, equal to K(br)/K(th) ratio. Yellow highlighted are significant *p*-values ($p < 0.05$).

Table S11. Summary table for comparing coefficients *D*, *K*, *MGEFDI* and *MGEI*.

**Breed (type)**	***D*(br)**	***D*(th)**	***K*(br) *N*[1:6]**	***K*(th) *N*[1:4]**	**MGEFDI = *D*(br)/*D*(th)**	**MGEI = *K*(br)/*K*(th)**	**FC (*GHR*)**	**BW1**	**BW14**	**BW28**
BR (meat)	0.39935 N[1:6]	0.95423 N[1:4]	0.011950	0.081557	0.418504972595705	0.146523290459433	6.63	47.5	305.0	1157
WC (meat)	1.28919 N[1:6]	11.2111 N[3:7]	0.03696	0.23305	0.1149922844	0.158592576700279	5.62	49.3	291.7	1287.5
PRW (dual purpose)	1.1474 N[1:6]/ 0.71703 N[1:4]	1.5086 N[1:4]	0.061148	0.07171	0.475294975473949	0.85271231348487	4.35	44.9	265.2	1058.4
YC (dual purpose)	4.1035 N[1:6]	3.3772 N[1:4]	0.3160	0.5841	1.215059812862727	0.54100325286766	69.1	39.0	101.3	240
BB (dual purpose)	1.7033 N[1:6]/ 2.9682 N[2:7]	2.1218 N[1:4]	0.11900	0.15094	1.398906588745405	0.788392738836624	31.78	38.2	107.5	241.7
OMF (dual purpose)	1.7867 N[1:6]/ 2.9835 N[2:6]	1.1325 N[1:4]	0.096146	0.05711	2.634437086092715	1.6835230257398	3.32	35.5	93.1	167.8
LR (egg)	3.1087 N[1:6]	1.8585 N[1:4]	0.069838	0.025535	1.672693032015066	2.73499118856472	4.79	42.4	79.8	222.4
UG (game)	3.7397 N[1:6]	0.63220 N[1:4]	0.29769	0.06069	5.915374881366656	4.90509144834404	2.51	41.3	92.4	223.7

D(br) and *D*(th), fractal dimension coefficients for breast and thigh muscles based on formula (2); *K*(br) and *K*(th), coefficients for breast and thigh muscles based on formula (1).

Table S12. Expression levels of myogenesis genes (fold change values) in the tissues of the breast and thigh muscles of E14 chick embryos on the example of the WC breed.

**Muscles**	**Genes***							
	TBP	***MSTN***	***GHR***	***MEF2C***	***MYOD1***	***MYOG***	***MYH1***	***MYF5***
	**1**	**2**	**3**	**4**	**5**	**6**	**7**	**8**
Breast	1	4.89	5.62	2.91	2.19	−4.32	−16.22	−685.02
Thigh	1	4.03	3.05	−1.69	−12.13	−4640.29	−335.46	−25531.63

* *TBP*, TATA-binding protein (internal control); *MSTN*, myostatin; *GHR*, growth hormone receptor; *MEF2C*, myocyte proliferation factor 2c; *MYOD1*, myogenic differentiation 1; *MYH1*, myosin; *MYOG*, myogenin; *MYF5*, myogenesis factor 5.

Table S13. Transformation of numerical indicators of fold change (FC) for gene expression in the breast muscles of WC chick embryos into Y- and X-coordinates of points representing genes in a fractal portrait.

**Indicators**	**Genes***							
	TBP	***MSTN***	***GHR***	***MEF2C***	***MYOD1***	***MYOG***	***MYH1***	***MYF5***
	**1**	**2**	**3**	**4**	**5**	**6**	**7**	**8**
FC	1	4.89	5.62	2.91	2.19	−4.32	−16.22	−685
LG**	0	−2.29	−2.49	−1.54	−1.13	2.11	4.02	9.42
Y-coordinate = LG − LG_{min}***	2.49	0.2	0	0.95	1.36	4.6	6.51	11.91
X- coordinate****	0.49	0.2	0	0.95	0.36	0.6	0.51	0.91

* The designation of genes is the same as in Table 2. ** LG is calculated by formula (6). *** Minimum value LG_{min} = LG_{GHR} = −2.49. **** X-coordinate is equal to the fractional part of the decimal number corresponding to the Y-coordinate.

Table S14. Correlation of the bioconsolidation index with the phenotypic characteristics of embryos and chicks of various chicken breeds.

Breed	Egg weight, g	Body weight of chicks, g			Content of NO metabolites in E7 embryos			*Ind*
		1 day	14 days	28 days	Nitrates	NODs	Sum	
Broiler	64.8	47.5	305.0	1157.0	145.4	3.3	148.7	0.38
White Cornish	65.8	49.3	291.7	1287.5	152.2	9.5	161.7	0.75
Plymouth Rock White	64.3	44.9	265.2	1058.4	0.0	141.8	145.0	0.48
Yurlov Crower	60.4	39.0	101.3	240.0	0.0	149.6	149.6	0.38
Brahma Buff	60.4	38.2	107.5	241.7	100.0	36.0	136.0	0.00
Orloff Mille Fleur	51.7	35.5	93.1	167.8	0.0	131.5	131.5	0.00
Layer	64.2	42.4	79.8	222.4	0.0	138.9	138.9	0.00
Uzbek Game	55.7	41.3	92.4	223.7	143.4	8.8	152.2	0.38
Standard error	±0.8	±3.1	±19.2	±50.0	±8.3	±9.4	±12.5	±0.03
Correlation coefficient with *Ind* (±0.02)	0.48	0.76	0.73	0.77	0.27	−0.17	0.89	1.00
Correlation coefficient with sum of NO metabolites (±0.02)	0.45	0.72	0.51	0.56	–	–	1.00	0.89
